# Origin matters: mycorrhizal growth response and induced resistance to pathogens depend on mycorrhizal and pathogen source

**DOI:** 10.1111/nph.70358

**Published:** 2025-07-06

**Authors:** Camille S. Delavaux, Haley Burrill, Robert Menning, Eric B. Duell, Reb L. Bryant, Terra Lubin, James D. Bever

**Affiliations:** ^1^ Institute of Integrative Biology, ETH Zurich (Swiss Federal Institute of Technology) Universitätsstrasse 16 8092 Zurich Switzerland; ^2^ Kansas Biological Survey and Center for Ecological Research The University of Kansas 106 Higuchi Hall, 2101 Constant Ave Lawrence KS 66047 USA; ^3^ Institute of Ecology and Evolution The University of Oregon 272 Onyx Bridge, 1318 Franklin Blvd Eugene OR 97403 USA

**Keywords:** arbuscular mycorrhizal fungi, fungal pathogens, invasion, mycorrhizal‐induced resistance, oomycetes, restoration

## Abstract

- Arbuscular mycorrhizal fungi (AMF) are critical to native plant community ecology and influence plant invasions. Research has focused on nutritional benefits of AMF, although evidence shows that they may also confer pathogen resistance. However, most such work has focused on agriculturally relevant plant species. Therefore, whether AMF confer pathogen resistance to *native* (wild) plant species, and impact of novel plant–microbial relationships on this benefit, remains understudied.
- We conducted a series of experiments measuring mycorrhizal‐induced resistance (MIR) to pathogens in native prairie plant species. We tested for pathogenicity across 69 field‐isolated fungi and oomycetes across five plant species. We then conducted experiments assessing growth response to native and non‐native AMF and pathogens in three plant species from native populations and milkweed (*Asclepias syriaca*) from native and postagricultural populations.
- We found evidence of MIR in milkweed. Moreover, we identified differential effects of AMF depending on plant species, with milkweed from native populations showing benefits from AMF. Finally, growth response was mediated by local adaptation, with matching AMF–pathogen origin strengthening responses.
- This work illustrates the importance of locally sourced AMF and plants to native plant ecology and suggests that pathogen resistance may be an important dimension of AMF benefit.

## Introduction

The majority of plant species world‐wide rely on arbuscular mycorrhizal fungi (AMF). These fungi provide host plants with limiting nutrients in exchange for carbon (Smith & Read, 2008), often improving plant growth (Hoeksema *et al*., 2018). Because of the ubiquity and impact of this symbiosis, there is an abundance of work focused on understanding these plant–mycorrhizal relationships and their consequences for plant community structure (van der Heijden *et al*., 1998; Vogelsang *et al*., 2006; Koziol & Bever, 2016; Bauer *et al*., 2018; Pedone‐Bonfim *et al*., 2018; Delavaux *et al*., 2021, 2024). In particular, work shows that restoration outcomes can benefit from reintroduction of native AMF, resulting in more diverse and productive communities with greater abundance of desirable late‐successional native plant species (Koziol & Bever, 2017; Neuenkamp *et al*., 2019; Vahter *et al*., 2020; Koziol *et al*., 2023). This improved understanding of native plant–mycorrhizal relationships has consequently provided valuable insights for effective restoration of degraded landscapes (Koziol *et al*., 2018), including limiting colonization by non‐native, invasive plant species (Lubin *et al*., 2019; Koziol *et al*., 2023). Despite the global relevance of AMF and evidence that these fungi provide additional non‐nutritional benefits (Song *et al*., 2011, 2013; Delavaux *et al*., 2017) – including resistance to pathogens – the role of mycorrhizal fungi in pathogen resistance in native plants remains underexplored.

Arbuscular mycorrhizal fungi have been shown to protect plants against pests and pathogens both below‐ and aboveground; this mycorrhizal‐induced resistance (MIR) can act both systemically, by priming plants to subsequent attack by modulating salicylic or jasmonic acid pathways, or locally via competition for space within roots (Jung *et al*., 2012). However, because most evidence of MIR is from agriculturally relevant plant species (Gallou *et al*., 2011; Campos‐Soriano *et al*., 2012; Song *et al*., 2013; Mustafa *et al*., 2017), our understanding of MIR in native plant species is scarce. Recent work has shown that MIR conferred by local, native AMF may be important for plant resistance to insect herbivory in grassland systems (Middleton *et al*., 2015), but no work has tested the importance of coevolved AMF and root pathogens to native plant MIR.

Although early‐successional plant species are not typically the target of restoration, they offer a powerful natural experiment that can inform potential changes in plant–AMF ecology. Native early‐successional plant species have repeatedly recolonized disturbed systems, events that can be leveraged to study (1) whether benefits from AMF are present and (2) whether they may evolve as they interact with novel AMF. Post‐agricultural landscapes are characterized by degraded microbial communities, including AMF communities (Herzberger *et al*., 2015; House & Bever, 2018), and early‐successional species – unlike their late‐successional counterparts – more readily recolonize these environments, exposing them to altered AMF communities. This natural recolonization mirrors the seeding or planting of species back into degraded systems during restorations. As degradation of AMF communities can also facilitate invasion by non‐native, invasive plant species (Stinson *et al*., 2006; Pringle *et al*., 2009; Vogelsang & Bever, 2009; Dickie *et al*., 2017) and non‐native plants can have similar dependencies on, and sensitivities to, AMF as early‐successional native species (Cheeke *et al*., 2019), studying how interactions between early‐successional native plant species and AMF change with anthropogenic degradation of the AMF community can also offer insights into plant invasion. Therefore, these early‐successional, native plant species – by interacting with both native and degraded AM fungal communities – offer a model system to assess cascading effects of novel plant–microbial interactions.

Recent work focusing on early‐successional native species suggests that the evolution of AMF response in native prairie species can occur relatively quickly, over the course of *c*. 50 yr (Delavaux & Bever, 2022). This research, conducted in the tallgrass prairie system of the Midwest United States, showed that native plant–AMF relationships shift as these species colonize (i.e. invade into) post‐agricultural landscapes and their associated degraded AMF communities. Across four native plant species, remnant plant populations responded most positively to native AMF inocula, while post‐agricultural plant populations responded most positively to novel non‐native AMF inocula. These results are consistent with local adaptation (Núñez‐Farfán & Schlichting, 2001; Kawecki & Ebert, 2004; Williams, 2018) of plants to their available fungi and suggest that the same native plant species may respond differently to AMF inocula when they recolonize or are planted into post‐agricultural sites. In the context of prairie restorations, this work suggests that seed source should be an important consideration when designing restoration. To reinstate native plant–AMF relationships, plant material and soil microbes should be as genetically similar as possible to local native populations. Moreover, this change in AMF benefit could have subsequent impacts on MIR in these post‐agricultural plant populations. As pathogen interactions influence plant diversity and community structure (Mills & Bever, 1998; Mordecai, 2011; Bever *et al*., 2015; Crawford *et al*., 2019; Wang *et al*., 2023), a better understanding of MIR in native prairie plants and how plant–AMF–pathogen relationships may evolve during recolonization of post‐agricultural sites is vital to designing successful restorations and understanding the consequences of novel plant–microbial interactions.

Here, we test for (1) MIR to locally isolated pathogens and (2) evolution of this benefit within native prairie plant species. We take advantage of the tallgrass prairie system and species in which previous work showed evidence for evolution of mycorrhizal response (Delavaux & Bever, 2022). We first isolated root‐associated fungi and oomycetes from the field and tested their pathogenicity across five native prairie plant species. Next, we conducted experiments to test whether the addition of AMF may reduce detrimental pathogen effects first across three of those prairie species, and then more extensively across *Asclepias syriaca* (common milkweed). Ultimately, we identified several pathogens, differential effects of mycorrhizal inocula origin (native or non‐native) depending on plant species, evidence of MIR in *A. syriaca*, and found that mycorrhizal growth response (MGR) is mediated by local adaptation of mycorrhizal fungi and pathogens. Together, this work highlights the importance of (1) mycorrhizal and pathogen origin in mediating early‐successional plant species growth responses and (2) pathogen resistance as a mycorrhizal benefit, providing insights for both improved prairie restorations and invasion into novel systems.

## Materials and Methods

We focused our research on native plant species found in both remnant and post‐agricultural sites in eastern Kansas (KS) and Missouri (MO, one site), USA. Remnant sites represent patches of remnant tallgrass prairie that have not been previously tilled; post‐agricultural sites represent abandoned agricultural sites experiencing natural recolonization over time. The remnant sites included three sites at the University of Kansas Field station (Rockefeller Prairie, 39.0°N, 95.2°W, KS, and Dogleg Prairie, 30.1°N, 95.2°W, KS), Prairie Nature Park Prairie (38.9°N, 95.2°W, KS), Kill Creek Prairie (38.9°N, 95.0°W), and Jerry Smith Prairie (38.9°N, 94.6°W, MO). The post‐agricultural sites included two sites at Perennial Agriculture Project farm in Lawrence, KS (39.0°N, 95.2°W, KS), two sites of the University of Kansas Field station (Welda Prairie, 38.2°N, 95.3°W, KS, and Plot 4010, 39.1°N, 95.2°W, KS), and the Rock Chalk Park walking trails (39.93°N, 95.33°W, KS). A map of sampling locations is presented in Supporting Information Fig. S1.

### Fungal and oomycete isolation and identification

From June to October 2019, roots of the five focal plant species were collected across several replicate populations representing remnant and disturbed populations (Rockefeller Prairie, Dogleg Prairie, Prairie Nature Park Prairie, The Land Institute, and Plot 4010). Sampling was conducted using a trowel to remove the entire plant root system. Following collection, a subset of randomly sampled fine roots was surface‐sterilized with bleach (10%) for 10 min and subsequently plated on fungal and oomycete specific media – bacto malt agar for fungi and pimaricin, ampicillin, rifampicin, and pentachloronitrobenzene (PARP) for oomycetes (Lane *et al*., 2012). Plates were inspected daily to replate (i.e. subculture) morphotypes. This continued for *c*. 12 months until pure cultures could be maintained.

We extracted DNA of each resulting morphotype subculture for Sanger sequencing. For each culture, we scraped a roughly 3‐mm‐diameter section of the growing culture from a plate. We used the PrepMan Ultra reagent (Thermo Fisher, Waltham, MA, USA) and 2.8‐mm stainless steel ball filled tubes (OPS Diagnostics, Lebanon, NJ, USA) for homogenization in a beadbeater, following the PrepMan Ultra instructions for DNA extraction. All extractions were sent to Genewiz (South Plainfield, NJ, USA) for PCR and Sanger sequencing of either their default internal transcribed spacer (ITS) primers for fungal cultures or our custom ITS primers (ITS300/ITS4) for oomycetes (Riit *et al*., 2016). Sanger sequencing was performed using a 3730xl DNA Analyzer (Applied Biosystems). Once raw data were generated, we assembled contigs (i.e. continuous sequences) using the Geneious software v.2021.2 (New Zealand). These contigs were then (1) run through BLASTn for identification on National Center for Biotechnology Information (NCBI) (Sayers *et al*., 2022) and (2) matched to the FUNGuild database (Nguyen *et al*., 2016) for guild assignment. A full table of the NCBI matches and FUNGuild assignment can be found in Table S1.

### Glasshouse experiments

**Fig. 1:**
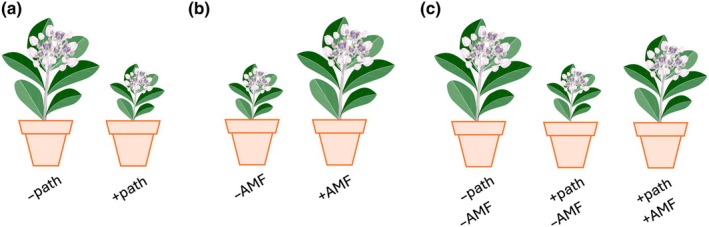
Conceptual representation of greenhouse experimental tests. Conceptual representation of (a) pathogenicity, (b) mycorrhizal benefit, and (c) mycorrhizal‐induced resistance (MIR) to pathogens. Pathogenicity occurs when there is reduced survival or growth when a plant is inoculated with a fungus or oomycete relative to sterile control. Mycorrhizal benefit occurs when there is increased growth when a plant is inoculated with arbuscular mycorrhizal fungi (AMF) relative to sterile control. Finally, MIR occurs when addition of AMF reduces negative effects of adding a pathogen.

We conducted four glasshouse experiments to test for fungal and oomycete pathogenicity, MGR and MIR to pathogens in tallgrass prairie species (Fig. 1). In the first two studies, we isolated fungi and oomycetes from our focal plant species and tested their pathogenicity on the target plants (2019–2020; each 42 d). In these first two experiments, we targeted five native early‐successional species: *Apocynum cannabinum* L. (Apocynaceae), *Vernonia fasciculata* L. (Asteraceae), *A. syriaca* L. (Apocynaceae), *Eupatorium altissimum* L. (Asteraceae), and *Solidago canadensis* L. (Asteraceae). In the third experiment (2021; 66 d), we tested for MGR, manipulating mycorrhizal population type (origin: remnant or post‐agricultural), and MIR across three plant species: *A. cannabinum*, *A. syriaca*, and *S. canadensis*. Finally, in the fourth experiment (2023; 74 d), we tested for MIR more intensively within *A. syriaca*, manipulating plant population type. All experiments were fully randomized (i.e. placed in blocks).

#### Pathogen inocula

Both fungal and oomycete inocula were created by using cultures (isolated as specified in the ‘Glasshouse experiments’ in the Materials and Methods section) stored on sterilized filter paper at −20°C. Fungal inocula were created by plating stored cultures on a potato dextrose agar and incubated at 32°C until approximately ⅔ plate coverage, between 3 and 6 d. Disk punches were taken using a ¼‐inch corer and replated, then incubated again under the same conditions. Disk punches were taken from these plates and added to 50‐ml vials of potato dextrose broth, then incubated at 32°C while agitated at 150 rpm for 10 d. Oomycete inocula were created by plating stored cultures on corn meal agar medium and incubated at 32°C for 5–10 d. Disk punches were taken using a ¼‐inch corer and replated, then incubated again under the same conditions. Fifty grains of sterilized, dry white rice was added to each plate, then incubated at 32°C for 7 d (Spear, 2015).

#### AMF inocula

We used two mixed AMF inocula to test differences in the AMF source. AMF species were chosen to represent the breadth of phylogenetic diversity in the respective community. We generated a native mix using AMF cultures originally collected from native prairie remnants in eastern Kansas to represent species typically found in local prairies (Koziol *et al*., 2020). Spores were separated from soil or trap cultures, grouped morphologically by species, inoculated, and propagated on native host plants following protocols available from the International Culture Collection of Vesicular Arbuscular Mycorrhizal Fungi (INVAM, University of Kansas). These cultures were maintained on a mixture of native prairie plant species, including *Schizachyrium scoparium*, *Dalea candida*, and *Liatris pycnostychya*. We generated a non‐native mix to represent species typically found in disturbed prairies (House & Bever, 2018) using AMF cultures. These cultures were propagated on *Sorghum bicolor* as part of the INVAM initiative. The native mix contained seven species: *Ambispora* sp., *Rhizophagus* sp., *Cetraspora pellucida*, *Gigaspora gigantea*, *Funneliformus mosseae*, *Glomus mortonii*, and *Claroidoglomus claroideum*. The non‐native (INVAM) mix contained 10 species: *Gigaspora gigantea* (from NC, USA), *Racocetra fulgida* (IN, USA), *Cetraspora pellucida* (Brazil), *Archaeospora trappei* (FL, USA), *Paraglomus occultum* (IA, USA), *Ambispora leptoticha* (IN, USA), *Ambispora gerdemannii* (MT, USA), *Rhizophagus clarus* (Brazil), *Septoglomus constrictum* (FL, USA), and *Funneliformus mosseae* (AZ, USA).

#### Pathogenicity experiments

For the first and second experiments, we tested for fungal and oomycete culture pathogenicity by measuring growth and survival in response to inoculation. We used seeds purchased from Prairie Moon Nursery (Winona, MN, USA). Each plant species was inoculated with each putative pathogen by direct addition of homogenized broth (50 ml) or rice (20 grains) to the root zone during planting (500‐ml pots). Plants were watered daily. For Experiment 1, each combination was replicated five times, with the sterile treatment (i.e. control: autoclaved broth or rice) of each plant species replicated five times for fungi and 10 times for oomycetes. For the Experiment 2, a subset of cultures from the first experiment that showed the strongest responses were tested. Here, each combination was replicated seven times, with the sterile treatment of each plant species replicated 10 times for fungi and 20 times for oomycetes. A detailed breakdown of each fungal and oomycete culture tested in which experiment is given in Table S1.

#### Mycorrhizal‐induced resistance experiments

For Experiments 3 and 4, we tested MGR and MIR to pathogens. For Experiment 3, both AMF inocula types (native and non‐native) were tested, but only using seeds from remnant sites (remnant) for three plant species: *A. cannabinum*, *A. syriaca*, and *S. canadensis*. For the Experiment 4, both AMF inocula types and both seed sources were tested (remnant and post‐agricultural) on *A. syriaca* alone. We selected *A. syriaca* for the final experiment due to both the accessibility of local seed populations and its disease incidence responses (MIR) in the previous experiment. For each species, seeds were hand‐collected from the five replicate remnant, and three post‐agricultural populations are detailed in the Materials and Methods section and shown in Fig. S1. Seeds were cleaned and cold‐stratified and taken out to germinate in sterile (i.e. autoclaved) perlite. After one month of germination, seedlings were planted in a sterilized 50 : 50 soil : sand mixture along with sterilized wide plastic straws so that the base of the straw reached the seedling root zone (1‐l pots). AMF inocula were added at planting. AMF inocula consisted of AMF communities (described in the ‘AMF inocula’ section) composed of root and soil cultures; for the sterile AMF treatment, inocula were omitted. Two weeks after planting, pathogen treatments (individual cultures) were added through the straws. Pathogen treatments received either 50 ml of cultured or sterile broth or 20 grains of cultured or sterile rice. We selected one fungal and two oomycete cultures to be used for these experiments, based on their impact on plant growth in previous experiments: fungal pathogen 35, oomycete pathogen 19, and oomycete pathogen 87. Plants were watered every 12 h.

Initial height was measured to account for initial plant size in our growth response assessments. At harvest, all plants were cut at the soil surface to separate above‐ and belowground biomass. Roots were manually washed to remove soil and other debris, then both above‐ and belowground biomass was dried at 70°C for one week. After sufficient drying, above‐ and belowground biomass were separately weighed. For the first AMF by pathogen experiment (Experiment 3), foliar disease incidence was measured. Measurements were taken on the severity of the disease: (0) no signs of disease, (1) leaf discoloration, (2) leaf discoloration and one spot, (3) two spots, (4) three to four spots, on multiple leaves, (5) > three leaves with spots, and (6) most leaves with spots (Fig. S2). Disease incidence was not measured in our second AMF by pathogen experiment (Experiment 4) as we could not visually distinguish between foliar disease and glasshouse arthropod herbivory. A subset of root tissue was taken at the time of harvest and placed in a plastic histological cassette for confirmation of AMF treatment success. Roots were cleared and subsequently stained with Trypan blue and glycerol, before confirmation of AMF colonization (Giovanetti & Mosse, 1980). We assessed a minimum of 20 slide intersects across three replicates per treatment by plant species.

### Statistical analyses

To investigate the effect of pathogens, AMF treatment, and/or their interactions on growth, survival, and disease, we use generalized linear mixed effect models. All models were inspected for homogeneity of variance. Next, contrasts were used to test specific comparisons within these models. We used the R packages tidyverse (Wickham *et al*., 2019), lme4 (Bates *et al*., 2015), lmertest (Kunzetsova *et al*., 2017), emmeans (Lenth *et al*., 2019), ggeffects (Lüdecke, 2018) for modeling, ggplot2 (Wickham, 2016) for visualization, and DHARMa (Hartig & Hartig, 2017) for model validation. Above‐ and belowground biomass were log‐transformed. All data and code can be found on the project Github repository (https://github.com/c383d893/MIRKS).

#### Pathogenicity in native prairie plant species

To test for pathogenicity (Experiments 1 and 2), we used models predicting aboveground biomass, belowground biomass, or survival. Independent variables included pathogen treatment (sterile or one of our cultures) as well as initial plant height. Models were run per plant species due to lack of replication not allowing for an interaction between species and culture (rank‐deficient models). Random effects included experimental block nested within experiment to control for the nonindependence of these factors. Models testing for biomass were Gaussian distribution, whereas those for survival were binomial.

#### Mycorrhizal fungi in native prairie plant species

Mycorrhizal treatment success was confirmed for both Experiments 3 and 4. To do this, we ran linear models predicting percent colonization, with AMF treatment and species (Experiment 3) or AMF treatment (Experiment 4) as independent variables. We subsequently ran contrasts to test for sterile vs both mycorrhizal treatments.

To test for mycorrhizal growth benefit (Experiments 3 and 4), we used models predicting aboveground biomass, belowground biomass, or survival. Independent variables included mycorrhizal treatment (sterile, native AMF, or non‐native AMF) as well as initial plant height. We also included an interaction between plant species and treatment. Random effects included experimental block nested within experiment to control for nonindependence of these factors. Models testing for biomass were Gaussian distribution, whereas those for survival were binomial. We subsequently used contrasts to test for differences between sterile vs both AMF treatments, sterile vs native AMF, sterile vs non‐native AMF, and native AMF vs non‐native AMF treatments within each plant species.

#### Mycorrhizal‐induced resistance to pathogens in native prairie plant species

To test for MIR (Experiments 3 and 4), we used models predicting above‐ and belowground biomass. Independent variables included mycorrhizal treatment (sterile, native, or non‐native AMF), pathogen treatment (sterile or one of our cultures), plant populations (remnant vs disturbed), and initial plant height. The aboveground model included an interaction between AMF and pathogen treatment, while the belowground model included an interaction between AMF and pathogen treatment and land use (the three‐way interaction was nonsignificant in the aboveground model). We subsequently used contrasts to test for differences between sterile vs both AMF treatments, sterile vs native AMF, sterile vs non‐native AMF treatments, and the interaction of pathogen sterile vs inoculated for each unique pathogen (19, 36, 87) by sterile vs native AMF and by sterile vs non‐native AMF.

## Results

### Pathogenicity of field‐isolated fungi and oomycetes

**Fig. 2:**
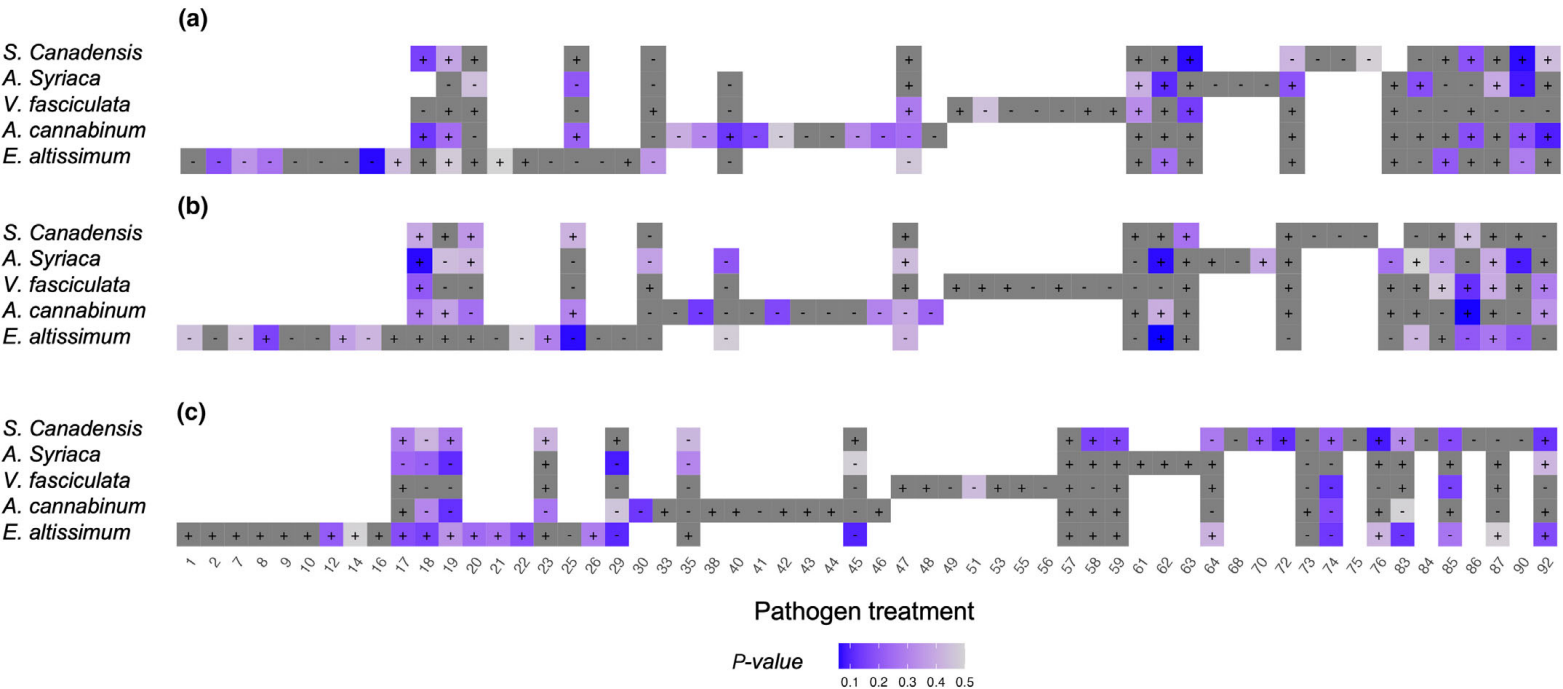
Above‐ and belowground biomass and probability of survival with putative pathogens. Plant species on y‐axis (*Solidago canadensis*, *Asclepias syriaca*, *Vernonia fasiculata*, *Apocynum cannabinum*, and *Eupatorium altissimum*), putative pathogen isolates on x‐axis. Blue shading denotes low *P*‐values, with gray representing nonsignificant differences from sterile plants. Plus (+) and minus (−) signs are based on the direction of the estimates: + indicates positive growth response, − denotes negative growth response, and thus pathogenicity. (a) Aboveground biomass response, (b) belowground biomass response, and (c) probability of survival.

We identified several fungal and oomycete cultures that acted as pathogens on our host plant species (total 9; Fig. 2; Table S1). Cultures were determined to be pathogenic when biomass or survival were reduced when plants were inoculated with these cultures relative to a sterile control. When assessing aboveground biomass, we found evidence of two pathogens, one each in *A. syriaca* (Culture 87, *P* = 0.08) and *E. altissimum* (Culture 14, *P* = 0.06). When assessing belowground biomass, we found evidence of three pathogens, one each in *A. syriaca* (Culture 87, *P* = 0.09) and *E. altissimum* (Culture 23, *P* = 0.07). Interestingly, Culture 19 led to increased aboveground growth in both *V. fasciculata* (*P* < 0.01) and *S. canadensis* (*P* < 0.001). Finally, when assessing survival (Table S2), we found evidence of six pathogens, one each in *A. syriaca* (Culture 29, *P* = 0.09) and *A. cannabinum* (Culture 64, *P* = 0.05), and four in *V. fasciculata* (Culture 29, *P* = 0.009; Culture 35, *P* = 0.008; Culture 45, *P* = 0.04; Culture 49, *P* = 0.05).

Sanger sequencing and BLAST identification revealed putative identification of these pathogens (Table S3). We further assessed putative guild via assignment to the FUNGuild database. Six out of eight could be matched; Cultures 14 and 23 could not be matched. Two cultures (64 and 87) had an assigned guild including ‘plant pathogen’ and were found to be in the genus *Fusarium*, known for pathogenic activity. Culture 19 was also identified as *Fusarium* sp. (a fungus), despite its positive effect on plant growth in our pathogenicity tests and its sustained growth on oomycete media. It therefore seems likely that this culture was not in fact an oomycete, but a fungus. Cultures 29 and 49 were also categorized as plant pathogens. Interestingly, Cultures 35 and 45 were assigned to the guilds of ‘fungal parasite’ and ‘undefined saprotroph’, respectively, highlighting the context dependency of pathogenicity and the potential limitations of applying these guild assignments.

### Mycorrhizal growth benefit across three native prairie species

Across both experiments including a mycorrhizal treatment, we found significantly higher colonization in treated relative to sterile controls (Experiment 3: *P* < 0.0001; Experiment 4: *P* = 0.01). All sterile controls had 0% colonization, except one with 5%.

**Fig. 3:**
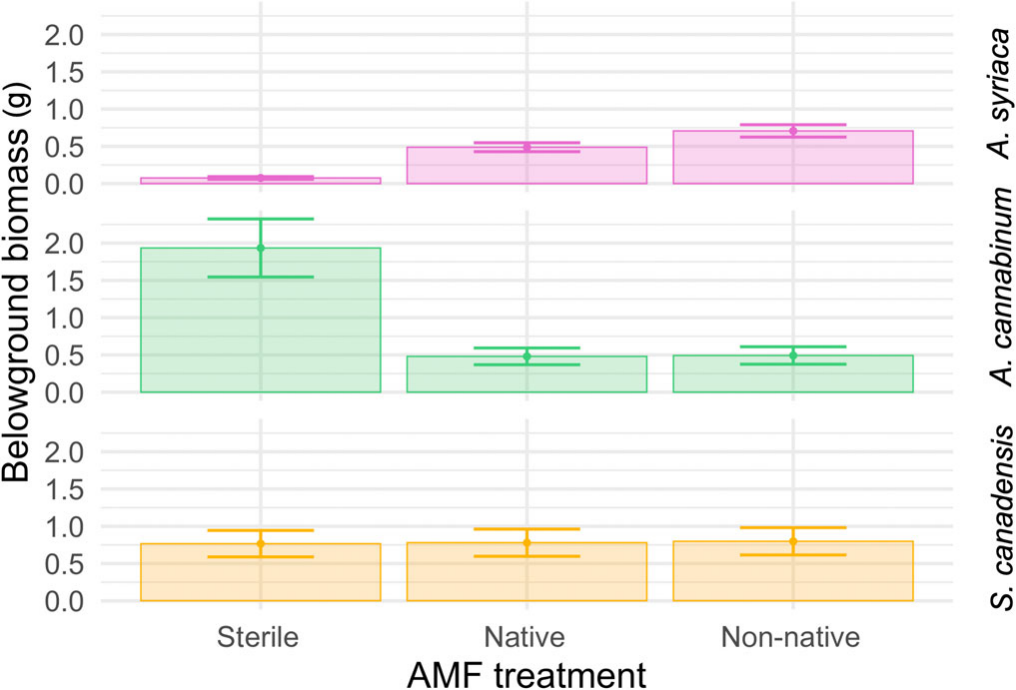
Mycorrhizal inocula source influences growth response in *Asclepias syriaca. Asclepias syriaca* responds positively to the addition of arbuscular mycorrhizal fungi, with biomass significantly higher for both AMF treatments, than for sterile (*P* < 0.0001). Moreover, *A. syriaca* responds significantly more positively to the addition of non‐native AMF relative to native AMF (*P* < 0.0001). *Apocynum cannabinum* responds negatively to the addition of either native or non‐native AMF (*P* < 0.0001), while *Solidago canadensis* does not respond to AMF addition (*P* = 0.9). Only belowground patterns are shown, but results are qualitatively similar for aboveground biomass (Supporting Information Fig. S3; Table S3). Error bars represent SE.

*Asclepias syriaca* showed positive MGR, producing greater biomass when inoculated with AMF compared with a sterile control (Figs 3, S3; Table S4). Moreover, MGR was higher when *A. syriaca* was grown with non‐native AMF relative to native AMF. This was consistent in above‐ and belowground biomass. Both above‐ and belowground biomass were significantly greater for both AMF treatments than for sterile (*P* < 0.0001) and greater with non‐native than with native AMF inocula (aboveground *P* < 0.01; belowground *P* < 0.0001). Specifically, *A. syriaca* produced on average 6.3 and 7.5 times more aboveground biomass with native and non‐native AMF inocula, respectively; *A. syriaca* produced on average 6.4 and 9.4 times more belowground biomass with native and non‐native AMF inocula, respectively.

The other two species tested – *A. cannabinum* and *S. canadensis* – showed contrasting patterns in response to AMF inoculation. *A. cannabinum* grew best without AMF inoculation, whereas *S. canadensis* showed no response to AMF inoculation. *A. cannabinum* aboveground biomass was highest in sterile compared with both AMF treatments (*P* < 0.0001), with non‐native AMF response significantly greater than native AMF (*P* = 0.01). This corresponds to 4.2 and 2.8 times *lower* aboveground biomass with native and non‐native AMF inocula compared with sterile. *A. cannabinum* belowground biomass mirrored aboveground patterns with biomass highest in sterile treatment (*P* < 0.0001); however, there was no difference between mycorrhizal treatments (*P* = 0.93). *S. canadensis* aboveground biomass showed no growth difference with AMF inoculation compared with sterile (*P* = 0.74) and no difference between mycorrhizal treatments (*P* = 0.37). Patterns were similar belowground with *S. canadensis* showing no response to AMF inoculation (*P* = 0.90) or AMF inoculation treatment (*P* = 0.93).

### Test of mycorrhizal‐induced resistance to pathogens in *A. syriaca*

**Fig. 4:**
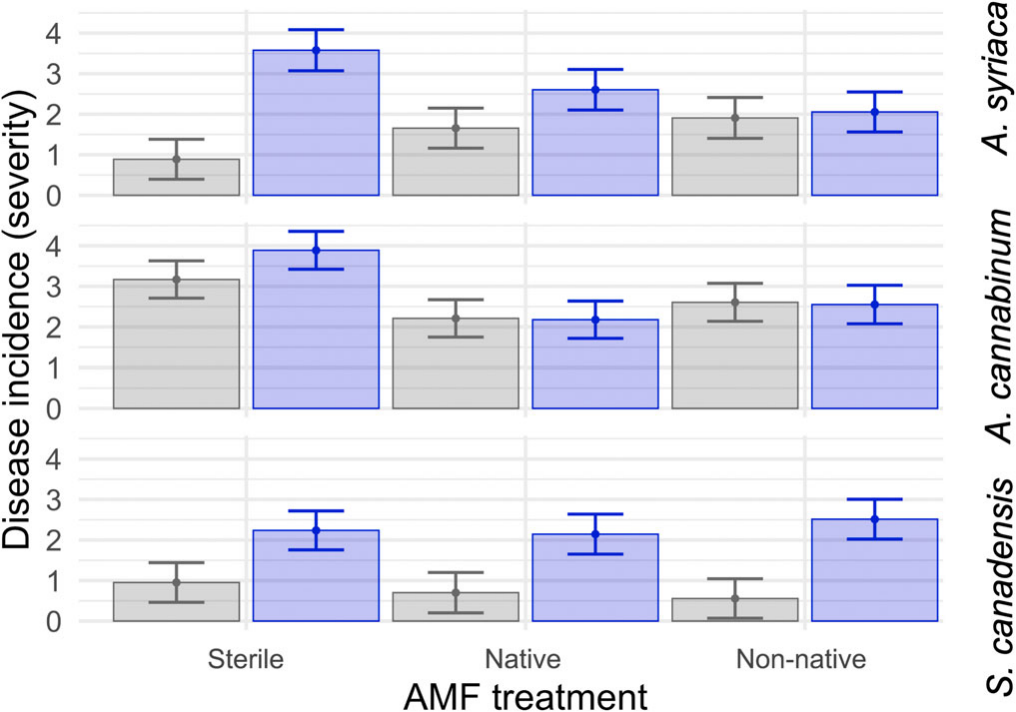
Evidence for mycorrhizal‐induced resistance in disease incidence in *Asclepias syriaca*. For *Asclepias syriaca*, disease incidence is reduced with AMF relative to sterile for both native AMF inocula and non‐native inocula (native *P* = 0.05; non‐native *P* < 0.01). This pattern is not found in *Apocynum cannabinum* or *Solidago canadensis* (Supporting Information Table S5). Blue indicates pathogen treatment; gray represents no pathogen treatment (i.e. sterile); error bars represent SE.

Patterns expected from MIR to pathogens emerged when investigating disease incidence (Fig. 4; Table S5). We found that disease incidence was reduced with AMF relative to sterile for both native and non‐native AMF inocula in *A. syriaca* (native *P* = 0.05; non‐native *P* < 0.01), confirming a role of AMF in protecting against pathogenic infection. We did not detect this pattern in *A. cannabinum* or *S. canadensis*.

**Fig. 5:**
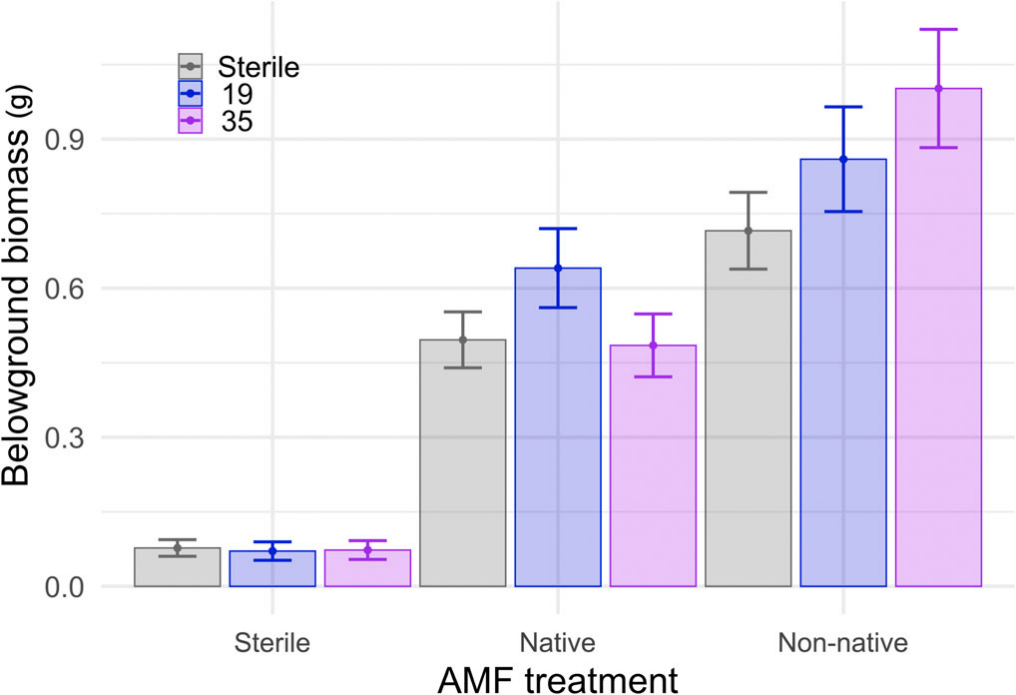
Arbuscular mycorrhizal fungal pathogen local adaption in *Asclepias syriaca. Asclepias syriaca* shows growth responses that depend on arbuscular mycorrhizal fungal (AMF)–pathogen population matching consistent with local adaptation. Specifically, when population types match (i.e. all native or both post‐agricultural), pathogen inoculation boots biomass relative to AMF sterile. Pathogen 19 was isolated from remnant prairie; 35 was isolated from post‐agricultural prairie. Error bars represent SE. Disease incidence was assessed based on the severity of the disease: (0) no signs of disease, (1) leaf discoloration, (2) leaf discoloration and one spot, (3) two spots, (4) three to four spots, on multiple leaves, (5) > 3 leaves with spots, and (6) most leaves with spots.

For both above‐ and belowground biomass, we found that AMF inoculation increased plant growth regardless of pathogen application (above‐ and belowground *P* < 0.0001). Neither pathogen suppressed plant growth under any condition. However, we did observe a significant interaction between pathogen source and AMF source (Fig. 5; Table S6). Specifically, when the source environment of the pathogen matched the source type of AMF (i.e. both native or both non‐native/post‐agricultural), pathogen inoculation increased biomass relative to that of the plant grown only with AMF. With non‐native AMF, Pathogen 35 – a post‐agricultural field‐isolated pathogen – increased biomass relative to AMF without pathogen (*P* < 0.01). Similarly, with native AMF, Pathogen 19 – a remnant isolated pathogen – increased biomass relative to AMF without pathogen (interaction pathogen sterile vs 19 by AMF sterile vs native *P* = 0.03). No significant results were found with Pathogen 87. Furthermore, no effect of seed origin was detected (Table S6).

## Discussion

Through these experiments, we have (1) isolated and tested the pathogenicity of field fungi and oomycetes associated with native prairie plant species, (2) detected a positive mycorrhizal response in the early‐successional species *A. syriaca*, (3) found evidence for MIR in disease incidence in *A. syriaca*, and (4) provided evidence of improved growth when pathogen and mutualist origins match. This series of experiments provides evidence of MIR using all wild components – including plant, pathogen, and mutualist material. Together, this work represents an important step in designing, testing, and finding evidence for the mycorrhizal benefit of pathogen resistance in native plant species. These results will inform improved ecological restorations and shed light on how plant–microbial interactions shape the success of reestablished or introduced plant species.

### Pathogenicity tests

Creating more realistic experiments requires using co‐occurring species. Here, we isolated fungi and oomycetes from plant species within our study sites. We then conducted pathogenicity tests on our study species for downstream use in our final two experiments. Although this takes time and effort, it allows for locally relevant inferences. Interestingly, taxa we identified as pathogens in our first two experiments did not always align with results from the most commonly used approach to guild assignment via FUNGuild. This mismatch highlights the limitations of current pathogen identification tools. FUNGuild and other similar databases are constructed from experimental tests or observations under limited conditions and host plants. It is therefore unavoidable that specific plant species and fungal taxa from a particular origin may deviate from guilds reported in such databases. Of the eight fungal and oomycete taxa that were pathogenic on one or several of our five target plant species, six were matched to the FUNGuild database, and four had ‘plant pathogen’ as a listed guild. One of our cultures (19) was identified as a *Fusarium* sp. but did not consistently act as a pathogen, even improving growth in certain species. In some cases, *Fusarium* species, particularly *F. equiseti*, are known to be beneficial to plant growth, inducing plant defenses against a broad range of antagonists, including root pathogens (Macia‐Vicente *et al*., 2008; Saldajeno & Hyakumachi, 2011; Pappas *et al*., 2018). Our work identifying pathogens in this tallgrass prairie system suggests that in order to make glasshouse experiments more realistic, investing in isolating local root‐associated taxa is worthwhile.

### Mycorrhizal‐induced resistance

We found evidence of MIR to pathogens in *A. syriaca* when assessing disease incidence. In particular, as expected from MIR, we found that foliar disease symptoms were suppressed with AMF inoculation (Fig. 5). Despite the relatively coarse method of disease incidence scoring, this result supports the protective role of AMF against pathogens. Importantly, this result is, to our knowledge, the first evidence of MIR to root pathogens in native prairie species collected from the field. This suggests that MIR may occur beyond more commonly tested agricultural plant species (Gallou *et al*., 2011; Campos‐Soriano *et al*., 2012; Mustafa *et al*., 2017) and more generally against diverse antagonists (Middleton *et al*., 2015), with implications for the functioning of AMF in native systems and potential benefit during restoration. By contrast, when we tested for MIR to pathogens in biomass, results were counter to our hypothesized MIR effect. Contrary to our expectations, we did not find that the pathogens suppressed plant growth under any condition. It is possible that the detected disease incidence scores indicate early signs of plant attack and that the length of our experiment did not allow enough time for plant biomass responses to the pathogens.

In our fourth experiment, we observed a positive growth response with pathogen inoculation, and an increase in this growth when plants were inoculated with AMF. Although initially counterintuitive, the specific glasshouse conditions at the time may explain this response. In particular, plants during the final MIR experiment experienced severe pressure from pest herbivores, including thrips and spider mites. It is possible that initial pathogen inoculation primed a defensive response by the plants, which in turn helped the plants resist the subsequent herbivores (Baldwin & Callahan, 1993; Pappas *et al*., 2018). We suggest that initial pathogens primed the plants and that subsequent glasshouse conditions were not favorable to their persistence. Plants inoculated with AMF, in particular, increased this pathogen priming of subsequent herbivore attack, and therefore benefited in growth. Unfortunately, we did not explicitly measure herbivore damage, so we could not directly test this hypothesis. Together, these results highlight the role of AMF in providing defense against antagonists.

### AMF–pathogen local adaptation

Our final study is consistent with local adaptation of plants with their associated microbes. We found support for the local adaptation (Núñez‐Farfán & Schlichting, 2001; Kawecki & Ebert, 2004; Williams, 2018) of pathogens and AMF from within the same land‐use history type in our fourth experiment with *A. syriaca*. To date, many studies have found evidence of local adaptation in the context of plant–pathogen (Thrall *et al*., 2002; Hoeksema & Forde, 2008; Cassetta *et al*., 2023) and plant–AMF (Johnson *et al*., 2010; Rúa *et al*., 2016; Bauer *et al*., 2020) interactions. Previous work has shown that locally adapted AMF improves resistance of native plants to herbivory (Middleton *et al*., 2015); however, to the best of our knowledge, our study is the first of its kind to demonstrate local adaptation between mutualists and pathogens across land‐use histories. We found that belowground growth patterns amplified when the origin of AMF and pathogen components matched. When pathogens and AMF shared a land‐use origin (remnant vs post‐agricultural), plants benefited from inoculation with pathogens. We found evidence of this for both matching native and non‐native (post‐agricultural for pathogens) components, with a native pathogen (19) increasing biomass when inoculated with native AMF and a post‐agricultural pathogen (35) increasing biomass when inoculated with non‐native AMF. This finding suggests that land‐use history has a significant impact on plant–microbial interactions and resulting fitness outcomes. Interestingly, plant origin did not significantly alter results, suggesting that microbial interactions may be more important than previously thought. It is also important to note that the AMF used in this experiment represent non‐native cultures that have not co‐evolved with recolonizing plant species in post‐agricultural sites. However, these were chosen to represent taxa known to occur in these sites (House & Bever, 2018). Future work should assess similar questions with AMF cultured directly from post‐agricultural sites to confirm local adaptation between all three components within these sites.

### Restoration implications

Our work informs restoration in several key ways. In testing early‐successional plant species, we find that AMF inoculation is detrimental to *A. cannabinum* and has no impact on *S. canadensis*, consistent with previous work finding that early‐successional plant species are generally less responsive than late‐successional species (Koziol & Bever, 2015; Cheeke *et al*., 2019). The reduced biomass of *A. cannabinum* with AMF inoculation highlights the context dependency of the mycorrhizal mutualism; the AMF tested here act antagonistically to this plant species. However, we do find that *A. syriaca* – an early‐successional species – responds positively to AMF. Together, the work presented here shows that AMF benefit is plant host‐specific, and some early‐successional species establishment may also benefit from co‐inoculation with AMF in restorations (Reynolds *et al*., 2020). Another major finding from our work is that AMF in grassland systems may provide benefits beyond nutrient uptake, including pathogen resistance. This emphasizes the potential multifunctionality of these key mutualists in restoration and supports accumulating evidence that remnant prairie microbe inoculation is critical for successful restoration (Koziol *et al*., 2020; Badger Hanson & Docherty, 2023). Future work should focus on the diversity of mycorrhizal benefits and native AMF communities that can supply these varied services.

### Implications for invasion

AMF impact on plant invasion is evident from studies at global (Delavaux *et al*., 2021) and local (Stinson *et al*., 2006; Pringle *et al*., 2009; Vogelsang & Bever, 2009; Dickie *et al*., 2017) scales, with most studies focusing on shifts in relative responsiveness or dependence of native and non‐native plant species. Although our research focused on native species from remnant relative to post‐agricultural populations, the methods and experimental designs used here could be applied to novel plant–microbial relationships present in invaded systems, or to compare between native, non‐native, and invasive plant species within an invaded site. Moreover, our results inform the selective forces experienced by colonizers of soils with degraded AMF communities, which in North America tend to be non‐native plant species (Seifert *et al*., 2009; Bauer *et al*., 2018). For example, we observed both here and in a previous study (Delavaux & Bever, 2022) evidence of evolutionary change in the populations of early‐successional native plant species during colonization of lands with degraded mycorrhizal communities. Similar results have been observed for non‐native plant species, as populations of *Hypericum perforatum* evolved reduced dependencies on AMF during invasion of degraded landscapes in North America (Siefert *et al*., 2009). While we do not currently have evidence that degraded AMF composition will alter AMF influence on pathogen protection for non‐native plant species, we do know that the defensive chemistry and tolerance to insect herbivory in the non‐native plant species, *Plantago lanceolata*, is highly sensitive to AMF composition (Bennett & Bever, 2007; Bennett *et al*., 2009). Given the potential importance of co‐evolution, we posit that native AMF would offer a greater degree of pathogen protection to native plant species than that of non‐native plant species. Future work should consider whether both direct nutritional and defense benefits differ between native and invasive plants, and how these benefits depend on whether the AMF are native or from anthropogenically degraded sites. As several of the early‐successional plant species which we found evolved to AMF degradation in North America are invasive in Asia and Europe, it would also be interesting to compare the native to introduced populations in their growth and defensive response to North American vs. Eurasian AMF communities.

### Conclusions

Collectively, this work uncovers important context dependencies of pathogenicity, response to mycorrhizal fungi, and MIR in tallgrass prairie plant species. Results show that certain native early‐successional plant species not only respond to mycorrhizal fungi but also exhibit some degree of mycorrhizal response specificity based on origin. Specifically, our results show that *A. syriaca* benefits from inoculation with AMF, particularly from non‐native AMF. In support of MIR, we find that there is reduced foliar disease when plants are inoculated with AMF. Finally, we find that there is local adaptation of response between mycorrhizal and pathogen components; that is, when these components share an origin, growth response patterns strengthen. These results highlight the need to integrate AMF in restoration as well as in invasion ecology.

## Competing interests

None declared.

## Author contributions

CSD, HB and JDB planned and designed the research. CSD, HB and RM performed experiments. CSD, JDB, EBD, RLB and TL conducted fieldwork. CSD led analyses with support from HB and JDB. CSD and HB wrote the manuscript with all other author contributing to writing. CSD and HB contributed equally to this work.

## Disclaimer

The New Phytologist Foundation remains neutral with regard to jurisdictional claims in maps and in any institutional affiliations.

## Supporting information


**Fig. S1** Map of sampling sites.
**Fig. S2** Disease incidence assessment.
**Fig. S3** Arbuscular mycorrhizal fungal inocula source influences growth response in *Asclepias syriaca*.
**Table S1** Pathogenicity growth results.
**Table S2** Pathogenicity survival results.

**Table S3** List of all tested fungal and oomycete taxa, field metadata, and associated sequences.
**Table S4** Arbuscular mycorrhizal fungal growth and survival.
**Table S5** Disease resistance.
**Table S6** Arbuscular mycorrhizal fungal and pathogen.
Please note: Wiley is not responsible for the content or functionality of any Supporting Information supplied by the authors. Any queries (other than missing material) should be directed to the *New Phytologist* Central Office.

## Data Availability

All data and code can be found on the project Github repository (https://github.com/c383d893/MIRKS). All raw sequence data can be found at NCBI project PRJNA1151050.
