# Supplementary material for: Origin matters: mycorrhizal growth response and induced resistance to pathogens depend on mycorrhizal and pathogen source
Source: New Phytol. 2025 Jul 6;248(3):1516–26. doi: 10.1111/nph.70358 (PMC12489296; doi:10.1111/nph.70358)
Supplement: Supplementary file 1 — Fig. S1 Map of sampling sites. Fig. S2 Disease incidence assessment. Fig. S3 Arbuscular mycorrhizal fungal inocula source influences growth response in Asclepias syriaca. Table S1 Pathogenicity growth results. Table S2 Pathogenicity survival results. [file NPH-248-1516-s002.zip › MIRKS_SI_July22025.docx]

**New Phytologist Supporting Information**
Article title: Origin matters: mycorrhizal growth response and induced resistance to pathogens depends on mycorrhizal and pathogen source
Authors: *Camille S. Delavaux, Haley Burrill, Robert Menning, Eric B. Duell, Reb L. Bryant, Terra Lubin, James D. Beve*

Article acceptance date: 17 June 2025

**Figure S1 | Map of sampling sites**

Map of sampling sites, including five remnant and five post-agricultural sites. All sites are located in Kansas, USA (KS) , with the exception of one site in western Missouri, USA (MO).

**
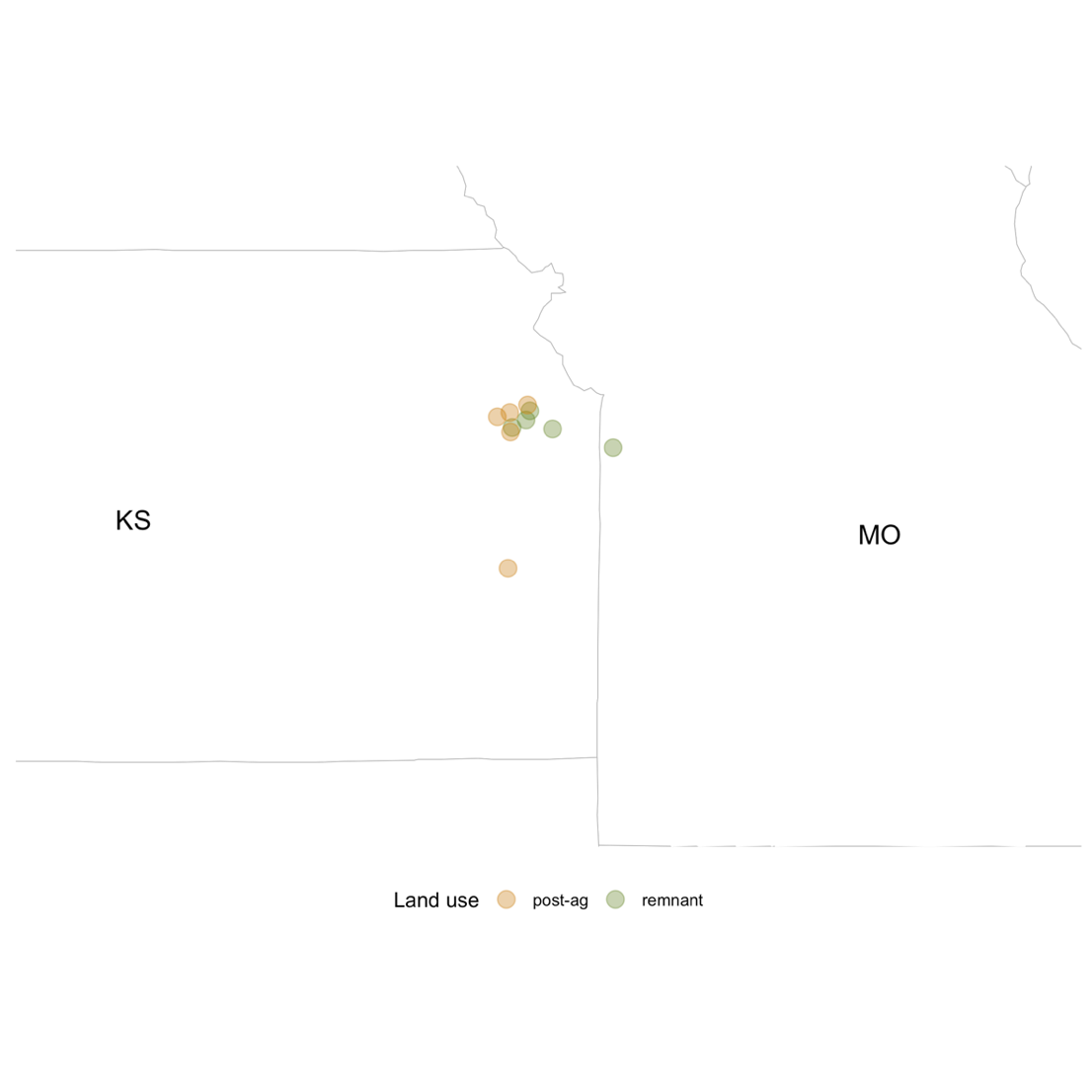
**

**Figure S2 | Disease incidence assessment**

Photos of example plant of disease incidence score 4, where there are >2 spots and on multiple

leaves.


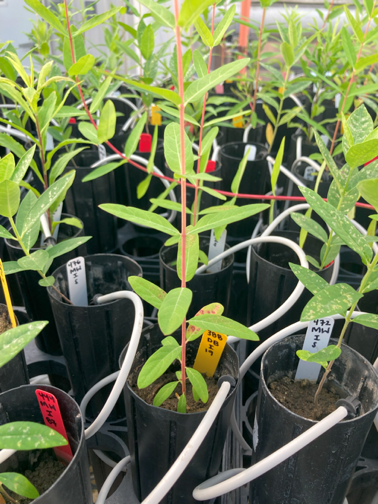

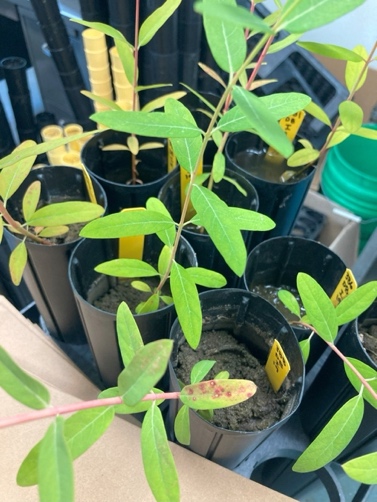


**Figure S3 | Arbuscular mycorrhizal fungal inocula source influences growth response in *Asclepias syriaca***

*Asclepias* *syriaca* responds positively to addition of arbuscular mycorrhizal fungi (AMF), with biomass significantly higher for both AMF treatments, compared to sterile (p < 0.0001). Moreover, *Asclepias* *syriaca* responds significantly more positively to addition of non-native AMF relative to native AMF (p < 0.001). *Apocynum cannabinum* responds negatively to the addition of either native or non-native AMF (p < 0.001) and responds marginally significantly more positively to addition of non-native AMF relative to native AMF (p = 0.013), while *Solidago canadensis* does not respond to AMF addition (p = 0.7). Error bars represent standard errors.


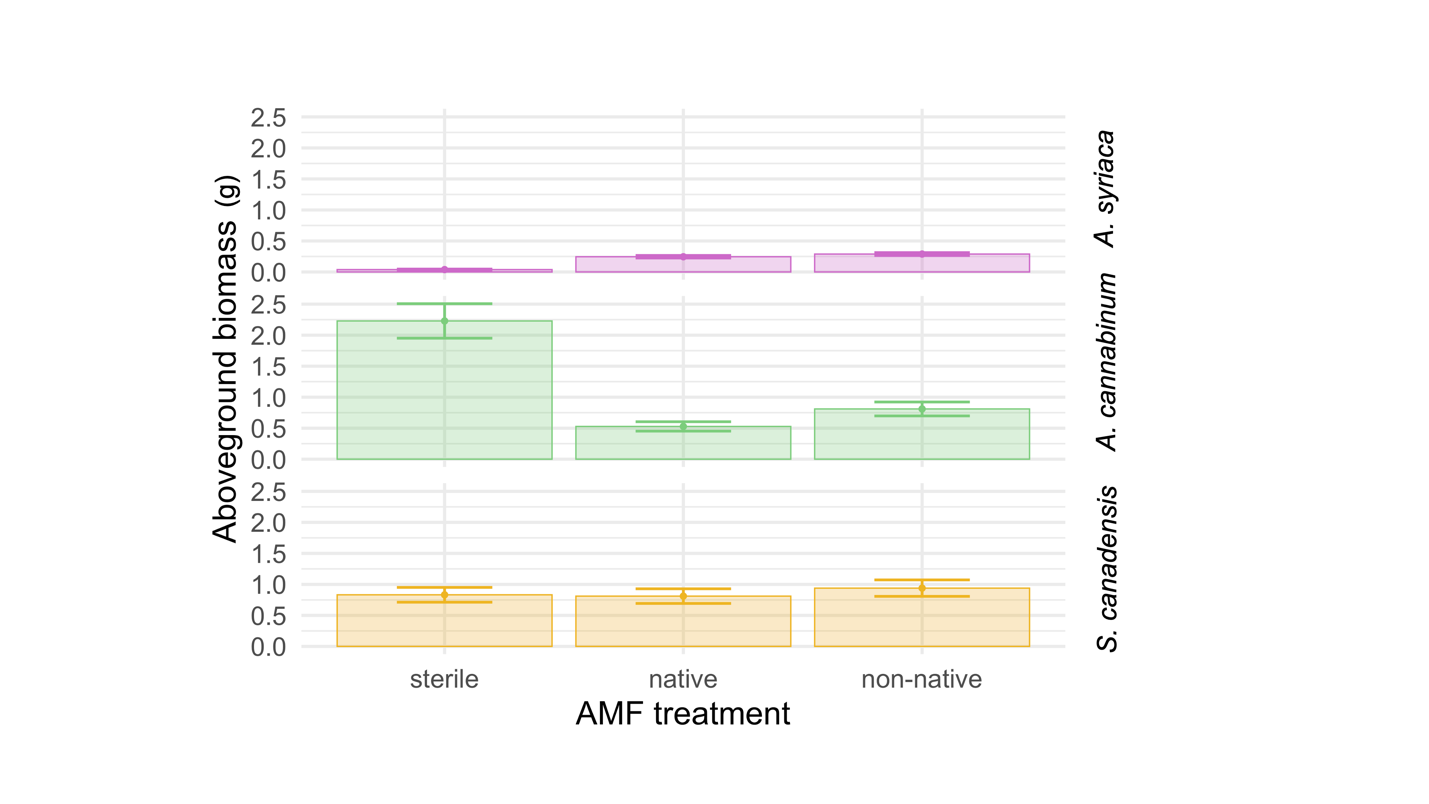


**Table S1 | Pathogenicity growth results**

Model outputs for pathogenicity tests on above- and belowground biomass; shown per plant species.

| **Aboveground** | | | | | |
| --- | --- | --- | --- | --- | --- |
| ***Asclepias syriaca*** | | | | | |
| variable | estimate | std. error | df | t value | p value |
| (Intercept) | -2.173 | 0.027 | 104 | -80.511 | <2.00E-16 |
| path_treat18 | -0.016 | 0.032 | 104 | -0.486 | 0.628 |
| path_treat19 | -0.027 | 0.036 | 104 | -0.758 | 0.450 |
| path_treat23 | -0.037 | 0.029 | 104 | -1.281 | 0.203 |
| path_treat29 | -0.016 | 0.061 | 104 | -0.257 | 0.798 |
| path_treat35 | -0.024 | 0.045 | 104 | -0.531 | 0.596 |
| path_treat45 | 0.003 | 0.032 | 104 | 0.089 | 0.929 |
| path_treat57 | 0.021 | 0.025 | 104 | 0.830 | 0.409 |
| path_treat58 | 0.043 | 0.027 | 104 | 1.610 | 0.110 |
| path_treat59 | 0.000 | 0.027 | 104 | 0.009 | 0.993 |
| path_treat61 | -0.008 | 0.030 | 104 | -0.257 | 0.798 |
| path_treat62 | -0.007 | 0.029 | 104 | -0.235 | 0.815 |
| path_treat63 | -0.007 | 0.031 | 104 | -0.215 | 0.831 |
| path_treat64 | 0.029 | 0.022 | 104 | 1.319 | 0.190 |
| path_treat73 | 0.022 | 0.037 | 104 | 0.598 | 0.551 |
| path_treat74 | 0.049 | 0.036 | 104 | 1.353 | 0.179 |
| path_treat76 | -0.016 | 0.032 | 104 | -0.507 | 0.613 |
| path_treat83 | -0.005 | 0.029 | 104 | -0.173 | 0.863 |
| path_treat85 | 0.021 | 0.025 | 104 | 0.852 | 0.396 |
| path_treat87 | -0.051 | 0.029 | 104 | -1.755 | 0.082 |
| path_treat92 | 0.001 | 0.027 | 104 | 0.053 | 0.958 |
| height_i | 0.010 | 0.007 | 104 | 1.402 | 0.164 |
| ***Apocynum cannabinum*** | | | | | |
| (Intercept) | -2.322 | 0.045 | 180 | -51.251 | <2.00E-16 |
| path_treat17 | 0.080 | 0.054 | 182 | 1.496 | 0.136 |
| path_treat18 | 0.065 | 0.056 | 174 | 1.154 | 0.250 |
| path_treat19 | -0.001 | 0.061 | 178 | -0.014 | 0.989 |
| path_treat23 | 0.068 | 0.056 | 162 | 1.207 | 0.229 |
| path_treat29 | -0.027 | 0.048 | 178 | -0.569 | 0.570 |
| path_treat30 | -0.057 | 0.068 | 168 | -0.835 | 0.405 |
| path_treat33 | -0.064 | 0.063 | 175 | -1.012 | 0.313 |
| path_treat35 | 0.072 | 0.048 | 179 | 1.517 | 0.131 |
| path_treat38 | -0.086 | 0.062 | 181 | -1.377 | 0.170 |
| path_treat40 | -0.045 | 0.063 | 173 | -0.715 | 0.476 |
| path_treat41 | -0.017 | 0.069 | 178 | -0.239 | 0.812 |
| path_treat42 | -0.026 | 0.061 | 175 | -0.421 | 0.674 |
| path_treat43 | -0.069 | 0.069 | 178 | -0.997 | 0.320 |
| path_treat44 | -0.076 | 0.063 | 177 | -1.212 | 0.227 |
| path_treat45 | -0.050 | 0.044 | 173 | -1.147 | 0.253 |
| path_treat46 | -0.019 | 0.062 | 182 | -0.307 | 0.760 |
| path_treat57 | 0.237 | 0.054 | 182 | 4.377 | 0.000 |
| path_treat58 | 0.119 | 0.054 | 182 | 2.216 | 0.028 |
| path_treat59 | 0.187 | 0.055 | 171 | 3.401 | 0.001 |
| path_treat64 | 0.018 | 0.061 | 178 | 0.296 | 0.768 |
| path_treat73 | 0.133 | 0.061 | 174 | 2.181 | 0.031 |
| path_treat74 | 0.166 | 0.069 | 181 | 2.414 | 0.017 |
| path_treat76 | 0.002 | 0.061 | 178 | 0.040 | 0.968 |
| path_treat83 | 0.077 | 0.057 | 182 | 1.336 | 0.183 |
| path_treat85 | 0.033 | 0.053 | 182 | 0.623 | 0.534 |
| path_treat87 | 0.074 | 0.057 | 181 | 1.305 | 0.194 |
| path_treat92 | 0.071 | 0.042 | 176 | 1.691 | 0.093 |
| height_i | 0.053 | 0.014 | 176 | 3.870 | 0.000 |
| ***Solidago canadensis*** | | | | | |
| (Intercept) | -2.278 | 0.017 | 86 | -130.535 | <2.00E-16 |
| path_treat17 | 0.041 | 0.028 | 86 | 1.439 | 0.154 |
| path_treat18 | 0.027 | 0.031 | 86 | 0.877 | 0.383 |
| path_treat19 | 0.112 | 0.027 | 86 | 4.099 | 0.000 |
| path_treat23 | 0.009 | 0.027 | 86 | 0.333 | 0.740 |
| path_treat29 | -0.005 | 0.034 | 86 | -0.144 | 0.886 |
| path_treat45 | 0.002 | 0.026 | 86 | 0.094 | 0.925 |
| path_treat57 | 0.056 | 0.025 | 86 | 2.290 | 0.024 |
| path_treat58 | 0.013 | 0.026 | 86 | 0.478 | 0.634 |
| path_treat59 | 0.049 | 0.026 | 86 | 1.859 | 0.067 |
| path_treat64 | -0.029 | 0.036 | 86 | -0.808 | 0.421 |
| path_treat68 | -0.017 | 0.056 | 86 | -0.297 | 0.767 |
| path_treat70 | -0.002 | 0.034 | 86 | -0.053 | 0.958 |
| path_treat72 | -0.021 | 0.030 | 86 | -0.694 | 0.490 |
| path_treat74 | -0.016 | 0.031 | 86 | -0.536 | 0.593 |
| path_treat76 | 0.005 | 0.028 | 86 | 0.161 | 0.872 |
| path_treat83 | 0.033 | 0.025 | 86 | 1.322 | 0.190 |
| path_treat85 | 0.090 | 0.035 | 86 | 2.575 | 0.012 |
| path_treat87 | 0.055 | 0.029 | 86 | 1.855 | 0.067 |
| path_treat92 | 0.020 | 0.026 | 86 | 0.768 | 0.445 |
| height_i | 0.020 | 0.016 | 86 | 1.278 | 0.205 |
| ***Eupatorium altissimum*** | | | | | |
| (Intercept) | -2.265 | 0.025 | 141 | -92.089 | <2.00E-16 |
| path_treat1 | -0.014 | 0.041 | 141 | -0.349 | 0.728 |
| path_treat10 | -0.003 | 0.037 | 141 | -0.089 | 0.929 |
| path_treat12 | -0.018 | 0.041 | 141 | -0.436 | 0.664 |
| path_treat14 | -0.075 | 0.041 | 141 | -1.857 | 0.065 |
| path_treat16 | 0.029 | 0.038 | 141 | 0.776 | 0.439 |
| path_treat17 | 0.070 | 0.028 | 141 | 2.519 | 0.013 |
| path_treat18 | 0.021 | 0.029 | 141 | 0.713 | 0.477 |
| path_treat19 | 0.010 | 0.030 | 141 | 0.341 | 0.734 |
| path_treat2 | -0.053 | 0.040 | 141 | -1.319 | 0.189 |
| path_treat20 | 0.028 | 0.041 | 141 | 0.677 | 0.500 |
| path_treat21 | 0.002 | 0.047 | 141 | 0.033 | 0.974 |
| path_treat22 | -0.003 | 0.041 | 141 | -0.072 | 0.943 |
| path_treat23 | -0.016 | 0.030 | 141 | -0.541 | 0.590 |
| path_treat25 | -0.014 | 0.077 | 141 | -0.182 | 0.856 |
| path_treat26 | 0.005 | 0.041 | 141 | 0.123 | 0.902 |
| path_treat29 | -0.072 | 0.077 | 141 | -0.934 | 0.352 |
| path_treat35 | -0.029 | 0.046 | 141 | -0.629 | 0.530 |
| path_treat45 | -0.057 | 0.078 | 141 | -0.727 | 0.469 |
| path_treat57 | 0.020 | 0.034 | 141 | 0.577 | 0.565 |
| path_treat58 | 0.036 | 0.032 | 141 | 1.135 | 0.258 |
| path_treat59 | 0.009 | 0.037 | 141 | 0.254 | 0.800 |
| path_treat64 | 0.000 | 0.034 | 141 | -0.004 | 0.996 |
| path_treat7 | -0.035 | 0.037 | 141 | -0.943 | 0.347 |
| path_treat73 | 0.001 | 0.056 | 141 | 0.020 | 0.984 |
| path_treat74 | -0.029 | 0.077 | 141 | -0.372 | 0.710 |
| path_treat76 | 0.051 | 0.041 | 141 | 1.264 | 0.208 |
| path_treat8 | -0.041 | 0.037 | 141 | -1.117 | 0.266 |
| path_treat83 | 0.010 | 0.056 | 141 | 0.177 | 0.860 |
| path_treat85 | 0.018 | 0.046 | 141 | 0.381 | 0.704 |
| path_treat87 | -0.039 | 0.037 | 141 | -1.066 | 0.288 |
| path_treat9 | -0.012 | 0.037 | 141 | -0.333 | 0.739 |
| path_treat92 | 0.007 | 0.034 | 141 | 0.212 | 0.832 |
| height_i | 0.053 | 0.013 | 141 | 3.995 | 0.000 |
| ***Vernonia fasciculata*** | | | | | |
| (Intercept) | -2.285 | 0.029 | 144 | -79.829 | <2.00E-16 |
| path_treat17 | -0.012 | 0.037 | 149 | -0.334 | 0.739 |
| path_treat18 | 0.002 | 0.039 | 148 | 0.041 | 0.967 |
| path_treat19 | 0.120 | 0.039 | 148 | 3.050 | 0.003 |
| path_treat23 | -0.005 | 0.037 | 149 | -0.137 | 0.891 |
| path_treat29 | 0.003 | 0.065 | 149 | 0.043 | 0.966 |
| path_treat35 | -0.033 | 0.091 | 149 | -0.367 | 0.714 |
| path_treat45 | 0.051 | 0.047 | 149 | 1.081 | 0.282 |
| path_treat47 | 0.000 | 0.044 | 148 | 0.000 | 1.000 |
| path_treat48 | -0.033 | 0.044 | 146 | -0.754 | 0.452 |
| path_treat49 | -0.006 | 0.054 | 149 | -0.103 | 0.918 |
| path_treat51 | -0.017 | 0.048 | 137 | -0.359 | 0.720 |
| path_treat53 | -0.028 | 0.043 | 149 | -0.654 | 0.514 |
| path_treat55 | 0.010 | 0.045 | 132 | 0.218 | 0.828 |
| path_treat56 | 0.001 | 0.048 | 124 | 0.025 | 0.980 |
| path_treat57 | 0.031 | 0.032 | 147 | 0.985 | 0.326 |
| path_treat58 | 0.004 | 0.032 | 149 | 0.130 | 0.896 |
| path_treat59 | 0.044 | 0.030 | 148 | 1.460 | 0.146 |
| path_treat64 | 0.017 | 0.037 | 149 | 0.475 | 0.636 |
| path_treat73 | 0.026 | 0.047 | 140 | 0.545 | 0.587 |
| path_treat74 | -0.002 | 0.054 | 149 | -0.043 | 0.966 |
| path_treat76 | -0.010 | 0.047 | 149 | -0.211 | 0.833 |
| path_treat83 | 0.013 | 0.037 | 148 | 0.359 | 0.720 |
| path_treat85 | -0.007 | 0.043 | 149 | -0.157 | 0.875 |
| path_treat87 | -0.008 | 0.037 | 149 | -0.212 | 0.833 |
| path_treat92 | -0.010 | 0.040 | 149 | -0.259 | 0.796 |
| height_i | 0.045 | 0.013 | 146 | 3.486 | 0.001 |
| **Belowground** | | | | | |
| ***Asclepias syriaca*** | | | | | |
| (Intercept) | -2.060 | 0.044 | 104 | -47.092 | <2.00E-16 |
| path_treat17 | 0.097 | 0.052 | 101 | 1.869 | 0.065 |
| path_treat18 | -0.039 | 0.051 | 95 | -0.771 | 0.443 |
| path_treat19 | 0.047 | 0.058 | 103 | 0.804 | 0.424 |
| path_treat23 | -0.019 | 0.047 | 105 | -0.407 | 0.685 |
| path_treat29 | -0.087 | 0.097 | 101 | -0.891 | 0.375 |
| path_treat35 | -0.092 | 0.072 | 105 | -1.281 | 0.203 |
| path_treat45 | 0.039 | 0.050 | 92 | 0.772 | 0.442 |
| path_treat57 | -0.001 | 0.040 | 99 | -0.023 | 0.981 |
| path_treat58 | 0.079 | 0.043 | 102 | 1.849 | 0.067 |
| path_treat59 | 0.020 | 0.043 | 101 | 0.460 | 0.647 |
| path_treat61 | 0.030 | 0.049 | 104 | 0.622 | 0.536 |
| path_treat62 | -0.015 | 0.046 | 99 | -0.322 | 0.748 |
| path_treat63 | 0.047 | 0.055 | 105 | 0.864 | 0.389 |
| path_treat64 | 0.005 | 0.035 | 105 | 0.152 | 0.879 |
| path_treat73 | -0.066 | 0.058 | 98 | -1.134 | 0.260 |
| path_treat74 | 0.039 | 0.058 | 100 | 0.678 | 0.500 |
| path_treat76 | -0.047 | 0.051 | 100 | -0.926 | 0.357 |
| path_treat83 | -0.029 | 0.050 | 81 | -0.593 | 0.555 |
| path_treat85 | 0.034 | 0.041 | 105 | 0.844 | 0.401 |
| path_treat87 | -0.082 | 0.047 | 103 | -1.722 | 0.088 |
| path_treat92 | 0.000 | 0.043 | 105 | -0.006 | 0.995 |
| height_i | 0.014 | 0.011 | 104 | 1.301 | 0.196 |
| ***Apocynum cannabinum*** | | | | | |
| (Intercept) | -2.238 | 0.030 | 174 | -74.252 | <2.00E-16 |
| path_treat17 | 0.038 | 0.036 | 173 | 1.066 | 0.288 |
| path_treat18 | 0.033 | 0.037 | 160 | 0.890 | 0.375 |
| path_treat19 | -0.046 | 0.041 | 169 | -1.138 | 0.257 |
| path_treat23 | 0.038 | 0.037 | 145 | 1.031 | 0.304 |
| path_treat29 | -0.021 | 0.032 | 173 | -0.675 | 0.501 |
| path_treat30 | -0.027 | 0.045 | 153 | -0.601 | 0.549 |
| path_treat33 | -0.064 | 0.043 | 179 | -1.486 | 0.139 |
| path_treat35 | -0.011 | 0.032 | 175 | -0.348 | 0.728 |
| path_treat38 | -0.092 | 0.042 | 180 | -2.208 | 0.029 |
| path_treat40 | -0.059 | 0.043 | 181 | -1.389 | 0.167 |
| path_treat41 | -0.011 | 0.046 | 172 | -0.230 | 0.819 |
| path_treat42 | -0.026 | 0.040 | 166 | -0.638 | 0.524 |
| path_treat43 | -0.015 | 0.047 | 181 | -0.323 | 0.747 |
| path_treat44 | -0.044 | 0.042 | 181 | -1.033 | 0.303 |
| path_treat45 | -0.025 | 0.029 | 159 | -0.857 | 0.393 |
| path_treat46 | -0.051 | 0.042 | 180 | -1.212 | 0.227 |
| path_treat57 | 0.131 | 0.036 | 178 | 3.608 | 0.000 |
| path_treat58 | 0.030 | 0.036 | 178 | 0.831 | 0.407 |
| path_treat59 | 0.140 | 0.037 | 172 | 3.739 | 0.000 |
| path_treat64 | 0.027 | 0.041 | 170 | 0.669 | 0.504 |
| path_treat73 | 0.110 | 0.040 | 163 | 2.733 | 0.007 |
| path_treat74 | 0.198 | 0.046 | 180 | 4.277 | 0.000 |
| path_treat76 | -0.014 | 0.041 | 170 | -0.336 | 0.737 |
| path_treat83 | 0.074 | 0.038 | 180 | 1.919 | 0.057 |
| path_treat85 | 0.007 | 0.036 | 181 | 0.200 | 0.841 |
| path_treat87 | -0.002 | 0.038 | 181 | -0.052 | 0.959 |
| path_treat92 | 0.026 | 0.028 | 165 | 0.947 | 0.345 |
| height_i | 0.042 | 0.009 | 166 | 4.578 | 0.000 |
| ***Solidago canadensis*** | | | | | |
| (Intercept) | -2.284 | 0.014 | 61 | -160.142 | <2.00E-16 |
| path_treat17 | 0.018 | 0.021 | 67 | 0.861 | 0.393 |
| path_treat18 | 0.014 | 0.021 | 67 | 0.639 | 0.525 |
| path_treat19 | 0.018 | 0.019 | 67 | 0.937 | 0.352 |
| path_treat23 | 0.016 | 0.020 | 67 | 0.814 | 0.419 |
| path_treat29 | -0.002 | 0.028 | 66 | -0.074 | 0.941 |
| path_treat35 | 0.015 | 0.038 | 64 | 0.391 | 0.697 |
| path_treat45 | 0.004 | 0.018 | 62 | 0.248 | 0.805 |
| path_treat57 | 0.003 | 0.017 | 67 | 0.158 | 0.875 |
| path_treat58 | 0.022 | 0.019 | 67 | 1.131 | 0.262 |
| path_treat59 | 0.000 | 0.018 | 67 | 0.015 | 0.988 |
| path_treat64 | -0.010 | 0.038 | 66 | -0.271 | 0.787 |
| path_treat68 | -0.008 | 0.038 | 66 | -0.220 | 0.827 |
| path_treat70 | -0.018 | 0.038 | 66 | -0.476 | 0.635 |
| path_treat74 | -0.015 | 0.028 | 66 | -0.525 | 0.601 |
| path_treat76 | 0.005 | 0.020 | 66 | 0.255 | 0.800 |
| path_treat83 | 0.016 | 0.021 | 66 | 0.767 | 0.446 |
| path_treat85 | 0.073 | 0.022 | 67 | 3.353 | 0.001 |
| path_treat87 | 0.050 | 0.020 | 65 | 2.453 | 0.017 |
| path_treat92 | -0.001 | 0.020 | 66 | -0.044 | 0.965 |
| height_i | 0.010 | 0.012 | 64 | 0.798 | 0.428 |
| ***Eupatorium altissimum*** | | | | | |
| (Intercept) | -2.273 | 0.011 | 134 | -201.621 | <2.00E-16 |
| path_treat1 | -0.014 | 0.018 | 134 | -0.755 | 0.451 |
| path_treat10 | -0.005 | 0.017 | 134 | -0.292 | 0.771 |
| path_treat12 | 0.016 | 0.018 | 134 | 0.892 | 0.374 |
| path_treat14 | -0.014 | 0.018 | 134 | -0.796 | 0.428 |
| path_treat16 | 0.000 | 0.017 | 134 | -0.022 | 0.983 |
| path_treat17 | 0.000 | 0.012 | 134 | -0.033 | 0.974 |
| path_treat18 | 0.033 | 0.014 | 134 | 2.437 | 0.016 |
| path_treat19 | 0.003 | 0.013 | 134 | 0.255 | 0.799 |
| path_treat2 | -0.013 | 0.020 | 134 | -0.648 | 0.518 |
| path_treat20 | -0.001 | 0.021 | 134 | -0.035 | 0.972 |
| path_treat21 | -0.015 | 0.021 | 134 | -0.715 | 0.476 |
| path_treat22 | 0.019 | 0.018 | 134 | 1.042 | 0.299 |
| path_treat23 | -0.026 | 0.014 | 134 | -1.851 | 0.066 |
| path_treat25 | -0.009 | 0.034 | 134 | -0.277 | 0.782 |
| path_treat26 | -0.007 | 0.018 | 134 | -0.412 | 0.681 |
| path_treat29 | -0.009 | 0.034 | 134 | -0.277 | 0.782 |
| path_treat35 | -0.014 | 0.021 | 134 | -0.701 | 0.484 |
| path_treat45 | -0.028 | 0.034 | 134 | -0.820 | 0.414 |
| path_treat57 | -0.001 | 0.015 | 134 | -0.091 | 0.928 |
| path_treat58 | 0.029 | 0.015 | 134 | 1.948 | 0.054 |
| path_treat59 | 0.008 | 0.016 | 134 | 0.506 | 0.614 |
| path_treat64 | -0.009 | 0.015 | 134 | -0.605 | 0.546 |
| path_treat7 | -0.012 | 0.016 | 134 | -0.744 | 0.458 |
| path_treat73 | -0.003 | 0.025 | 134 | -0.112 | 0.911 |
| path_treat74 | -0.027 | 0.034 | 134 | -0.795 | 0.428 |
| path_treat76 | 0.004 | 0.018 | 134 | 0.231 | 0.817 |
| path_treat8 | 0.023 | 0.016 | 134 | 1.425 | 0.156 |
| path_treat83 | -0.031 | 0.025 | 134 | -1.276 | 0.204 |
| path_treat85 | 0.022 | 0.020 | 134 | 1.094 | 0.276 |
| path_treat87 | -0.023 | 0.018 | 134 | -1.273 | 0.205 |
| path_treat9 | -0.003 | 0.016 | 134 | -0.186 | 0.853 |
| path_treat92 | -0.001 | 0.015 | 134 | -0.033 | 0.974 |
| height_i | 0.015 | 0.006 | 134 | 2.560 | 0.012 |
| ***Vernonia fasciculata*** | | | | | |
| (Intercept) | -2.271 | 0.033 | 139 | -69.465 | <2.00E-16 |
| path_treat17 | 0.054 | 0.042 | 148 | 1.274 | 0.205 |
| path_treat18 | -0.021 | 0.045 | 145 | -0.464 | 0.643 |
| path_treat19 | -0.024 | 0.045 | 144 | -0.544 | 0.587 |
| path_treat23 | -0.010 | 0.042 | 147 | -0.244 | 0.808 |
| path_treat29 | 0.032 | 0.074 | 148 | 0.435 | 0.665 |
| path_treat35 | -0.023 | 0.103 | 146 | -0.226 | 0.821 |
| path_treat45 | 0.402 | 0.054 | 148 | 7.488 | 0.000 |
| path_treat47 | 0.006 | 0.050 | 146 | 0.126 | 0.900 |
| path_treat48 | 0.031 | 0.050 | 141 | 0.618 | 0.538 |
| path_treat49 | 0.013 | 0.061 | 148 | 0.214 | 0.831 |
| path_treat51 | -0.004 | 0.054 | 126 | -0.080 | 0.936 |
| path_treat53 | 0.017 | 0.048 | 148 | 0.355 | 0.723 |
| path_treat55 | -0.013 | 0.051 | 118 | -0.264 | 0.793 |
| path_treat56 | -0.011 | 0.056 | 108 | -0.190 | 0.850 |
| path_treat57 | -0.015 | 0.036 | 142 | -0.419 | 0.676 |
| path_treat58 | -0.005 | 0.036 | 148 | -0.126 | 0.900 |
| path_treat59 | 0.009 | 0.036 | 148 | 0.260 | 0.795 |
| path_treat64 | 0.009 | 0.042 | 147 | 0.203 | 0.839 |
| path_treat73 | 0.013 | 0.054 | 130 | 0.235 | 0.815 |
| path_treat74 | 0.000 | 0.061 | 148 | -0.004 | 0.997 |
| path_treat76 | 0.039 | 0.054 | 148 | 0.730 | 0.467 |
| path_treat83 | 0.065 | 0.042 | 144 | 1.551 | 0.123 |
| path_treat85 | 0.041 | 0.048 | 148 | 0.840 | 0.402 |
| path_treat87 | 0.007 | 0.042 | 148 | 0.176 | 0.860 |
| path_treat92 | 0.048 | 0.045 | 147 | 1.053 | 0.294 |
| height_i | 0.032 | 0.015 | 143 | 2.163 | 0.032 |

**Table S2 | Pathogenicity survival results**

Model outputs for pathogenicity tests on survival; shown per plant species.

| **Survival** | | | | |
| --- | --- | --- | --- | --- |
| ***Asclepias syriaca*** | | | | |
| variable | estimate | std. error | z value | p value |
| (Intercept) | -0.631 | 0.866 | -0.728 | 0.466 |
| path_treat17 | -1.036 | 0.879 | -1.179 | 0.238 |
| path_treat18 | -1.083 | 0.884 | -1.225 | 0.221 |
| path_treat19 | -1.378 | 0.867 | -1.591 | 0.112 |
| path_treat23 | 0.001 | 0.937 | 0.002 | 0.999 |
| path_treat29 | -2.061 | 1.200 | -1.717 | 0.086 |
| path_treat35 | -1.023 | 1.013 | -1.010 | 0.312 |
| path_treat45 | -0.594 | 0.856 | -0.694 | 0.488 |
| path_treat57 | 19.030 | 8350.000 | 0.002 | 0.998 |
| path_treat58 | 0.746 | 1.159 | 0.644 | 0.520 |
| path_treat59 | 0.698 | 1.157 | 0.603 | 0.547 |
| path_treat61 | 18.030 | 8202.000 | 0.002 | 0.998 |
| path_treat62 | 18.660 | 9163.000 | 0.002 | 0.998 |
| path_treat63 | 16.320 | 3741.000 | 0.004 | 0.997 |
| path_treat64 | 0.462 | 0.875 | 0.528 | 0.597 |
| path_treat73 | -0.185 | 1.012 | -0.183 | 0.855 |
| path_treat74 | -0.270 | 1.023 | -0.264 | 0.792 |
| path_treat76 | 0.693 | 1.202 | 0.577 | 0.564 |
| path_treat83 | 0.030 | 0.929 | 0.032 | 0.974 |
| path_treat85 | 19.430 | 11140.000 | 0.002 | 0.999 |
| path_treat87 | 0.151 | 0.940 | 0.161 | 0.872 |
| path_treat92 | 0.917 | 1.157 | 0.792 | 0.428 |
| height_i | 0.470 | 0.233 | 2.020 | 0.043 |
| ***Apocynum cannabinum*** | | | | |
| (Intercept) | -0.399 | 1.172 | -0.340 | 0.734 |
| path_treat17 | 21.460 | 81950.000 | 0.000 | 1.000 |
| path_treat18 | -1.272 | 1.266 | -1.005 | 0.315 |
| path_treat19 | -1.606 | 1.034 | -1.553 | 0.120 |
| path_treat23 | -1.409 | 1.282 | -1.099 | 0.272 |
| path_treat29 | -0.916 | 1.248 | -0.734 | 0.463 |
| path_treat30 | -2.040 | 1.356 | -1.505 | 0.132 |
| path_treat33 | 21.080 | 91820.000 | 0.000 | 1.000 |
| path_treat35 | -0.705 | 1.229 | -0.574 | 0.566 |
| path_treat38 | 21.390 | 88260.000 | 0.000 | 1.000 |
| path_treat40 | 21.980 | 86010.000 | 0.000 | 1.000 |
| path_treat41 | -0.093 | 1.296 | -0.072 | 0.943 |
| path_treat42 | 22.730 | 107700.000 | 0.000 | 1.000 |
| path_treat43 | 21.330 | 99100.000 | 0.000 | 1.000 |
| path_treat44 | 21.240 | 73750.000 | 0.000 | 1.000 |
| path_treat45 | -0.140 | 1.218 | -0.115 | 0.909 |
| path_treat46 | 21.800 | 77060.000 | 0.000 | 1.000 |
| path_treat57 | 21.680 | 105000.000 | 0.000 | 1.000 |
| path_treat58 | 23.880 | 133000.000 | 0.000 | 1.000 |
| path_treat59 | 21.330 | 87130.000 | 0.000 | 1.000 |
| path_treat64 | -2.130 | 1.072 | -1.986 | 0.047 |
| path_treat73 | 21.390 | 93070.000 | 0.000 | 1.000 |
| path_treat74 | -1.807 | 1.321 | -1.368 | 0.171 |
| path_treat76 | 22.100 | 118600.000 | 0.000 | 1.000 |
| path_treat83 | -0.844 | 1.237 | -0.683 | 0.495 |
| path_treat85 | 22.450 | 95150.000 | 0.000 | 1.000 |
| path_treat87 | -0.442 | 1.239 | -0.356 | 0.722 |
| path_treat92 | 28.660 | 1709000.000 | 0.000 | 1.000 |
| height_i | 1.071 | 0.438 | 2.443 | 0.015 |
| ***Solidago canadensis*** | | | | |
| (Intercept) | -3.711 | 0.841 | -4.413 | 0.000 |
| path_treat17 | 1.719 | 1.641 | 1.047 | 0.295 |
| path_treat18 | -1.030 | 1.280 | -0.805 | 0.421 |
| path_treat19 | 2.007 | 1.862 | 1.078 | 0.281 |
| path_treat23 | 1.307 | 1.627 | 0.803 | 0.422 |
| path_treat29 | 0.804 | 1.296 | 0.620 | 0.535 |
| path_treat35 | -1.419 | 1.794 | -0.791 | 0.429 |
| path_treat45 | 3.055 | 1.502 | 2.034 | 0.042 |
| path_treat57 | 16.800 | 1844.000 | 0.009 | 0.993 |
| path_treat58 | 2.470 | 1.750 | 1.411 | 0.158 |
| path_treat59 | 2.237 | 1.621 | 1.380 | 0.168 |
| path_treat64 | -1.389 | 1.282 | -1.083 | 0.279 |
| path_treat68 | -1.193 | 1.883 | -0.633 | 0.526 |
| path_treat70 | 1.837 | 1.448 | 1.269 | 0.204 |
| path_treat72 | 2.426 | 1.549 | 1.566 | 0.117 |
| path_treat73 | -22.130 | 67330.000 | 0.000 | 1.000 |
| path_treat74 | 1.221 | 1.014 | 1.204 | 0.229 |
| path_treat75 | -6.164 | 16.500 | -0.374 | 0.709 |
| path_treat76 | 1.669 | 0.970 | 1.721 | 0.085 |
| path_treat83 | 1.242 | 1.252 | 0.992 | 0.321 |
| path_treat84 | -22.310 | 320100.000 | 0.000 | 1.000 |
| path_treat85 | -2.053 | 1.515 | -1.355 | 0.175 |
| path_treat86 | -9.884 | 16.490 | -0.599 | 0.549 |
| path_treat87 | -0.474 | 1.263 | -0.375 | 0.708 |
| path_treat90 | -91.980 | 30010000.000 | 0.000 | 1.000 |
| path_treat92 | 2.819 | 1.850 | 1.524 | 0.128 |
| height_i | 5.018 | 1.108 | 4.527 | 0.000 |
| ***Eupatorium altissimum*** | | | | |
| (Intercept) | -2.280 | 0.663 | -3.440 | 0.001 |
| path_treat1 | 0.108 | 1.198 | 0.090 | 0.928 |
| path_treat10 | 25.590 | 163000.000 | 0.000 | 1.000 |
| path_treat12 | 1.516 | 1.206 | 1.256 | 0.209 |
| path_treat14 | 0.830 | 1.218 | 0.681 | 0.496 |
| path_treat16 | 26.500 | 215600.000 | 0.000 | 1.000 |
| path_treat17 | 1.203 | 0.893 | 1.348 | 0.178 |
| path_treat18 | 1.240 | 0.871 | 1.424 | 0.154 |
| path_treat19 | 0.738 | 0.774 | 0.954 | 0.340 |
| path_treat2 | 0.315 | 1.192 | 0.265 | 0.791 |
| path_treat20 | 1.443 | 1.241 | 1.163 | 0.245 |
| path_treat21 | 1.201 | 1.092 | 1.100 | 0.271 |
| path_treat22 | 1.648 | 1.244 | 1.324 | 0.185 |
| path_treat23 | 0.576 | 0.862 | 0.668 | 0.504 |
| path_treat25 | -0.707 | 1.275 | -0.554 | 0.579 |
| path_treat26 | 1.331 | 1.250 | 1.065 | 0.287 |
| path_treat29 | -1.966 | 1.214 | -1.619 | 0.105 |
| path_treat35 | 0.215 | 1.096 | 0.196 | 0.844 |
| path_treat45 | -1.933 | 1.161 | -1.665 | 0.096 |
| path_treat57 | 31.340 | 5050000.000 | 0.000 | 1.000 |
| path_treat58 | 27.800 | 727500.000 | 0.000 | 1.000 |
| path_treat59 | 0.012 | 0.968 | 0.012 | 0.991 |
| path_treat64 | 1.074 | 1.315 | 0.816 | 0.414 |
| path_treat7 | 25.200 | 157900.000 | 0.000 | 1.000 |
| path_treat73 | -0.262 | 1.036 | -0.253 | 0.801 |
| path_treat74 | -1.839 | 1.232 | -1.493 | 0.135 |
| path_treat76 | 1.150 | 1.440 | 0.798 | 0.425 |
| path_treat8 | 25.490 | 287400.000 | 0.000 | 1.000 |
| path_treat83 | -1.473 | 0.969 | -1.520 | 0.129 |
| path_treat85 | -1.049 | 0.951 | -1.103 | 0.270 |
| path_treat87 | 0.703 | 1.015 | 0.693 | 0.489 |
| path_treat9 | 24.700 | 155100.000 | 0.000 | 1.000 |
| path_treat92 | 1.604 | 1.179 | 1.361 | 0.174 |
| height_i | 2.258 | 0.442 | 5.114 | 0.000 |
| ***Vernonia fasciculata*** | | | | |
| (Intercept) | 0.692 | 1.051 | 0.659 | 0.510 |
| path_treat17 | 23.777 | 762.728 | 0.031 | 0.975 |
| path_treat18 | -0.616 | 1.212 | -0.508 | 0.611 |
| path_treat19 | -0.612 | 1.216 | -0.504 | 0.614 |
| path_treat23 | 23.663 | 815.240 | 0.029 | 0.977 |
| path_treat29 | -2.813 | 1.076 | -2.614 | 0.009 |
| path_treat35 | -3.327 | 1.252 | -2.658 | 0.008 |
| path_treat45 | -1.939 | 0.949 | -2.044 | 0.041 |
| path_treat47 | 22.808 | 836.386 | 0.027 | 0.978 |
| path_treat48 | 23.129 | 836.386 | 0.028 | 0.978 |
| path_treat49 | -2.154 | 1.086 | -1.983 | 0.047 |
| path_treat51 | -0.972 | 1.258 | -0.772 | 0.440 |
| path_treat53 | 23.824 | 812.670 | 0.029 | 0.977 |
| path_treat55 | 23.931 | 844.814 | 0.028 | 0.977 |
| path_treat56 | -0.090 | 1.331 | -0.068 | 0.946 |
| path_treat57 | 0.074 | 1.182 | 0.063 | 0.950 |
| path_treat58 | -0.610 | 0.944 | -0.646 | 0.518 |
| path_treat59 | 25.524 | 812.670 | 0.031 | 0.975 |
| path_treat64 | 24.196 | 832.807 | 0.029 | 0.977 |
| path_treat73 | -0.573 | 1.258 | -0.456 | 0.648 |
| path_treat74 | -1.662 | 1.069 | -1.556 | 0.120 |
| path_treat76 | -0.500 | 1.260 | -0.397 | 0.691 |
| path_treat83 | 23.957 | 911.947 | 0.026 | 0.979 |
| path_treat85 | -1.439 | 1.007 | -1.429 | 0.153 |
| path_treat87 | 23.761 | 1044.086 | 0.023 | 0.982 |
| path_treat92 | -0.636 | 1.218 | -0.522 | 0.602 |
| height_i | 0.836 | 0.513 | 1.630 | 0.103 |

**Table S3 | List of all tested fungal and oomycete taxa, field metadata, and associated sequences**

Comprehensive list of tested field-collected fungi and oomycete tested in pathogenicity tests and associated metadata, including original population type (R is remnant, OF is old field, or post-agricultural), whether the isolate was sequences, putative species, what experiment it was included in, and FUNGuild derived guild. Uploaded as separate excel file.

**Table S4 | Arbuscular mycorrhizal fungal growth and survival**

Model outputs for above- and belowground biomass and survival with associated custom contrasts; shown per plant species.

| **Aboveground** | | | | | |
| --- | --- | --- | --- | --- | --- |
| variable | estimate | std. error | df | t value | p value |
| (Intercept) | 0.524 | 0.130 | 149.000 | 4.037 | 0.000 |
| AM_treatnative | -1.310 | 0.148 | 434.788 | -8.830 | <2.00E-16 |
| AM_treatnon-native | -0.940 | 0.149 | 432.426 | -6.324 | 0.000 |
| plantspA.syriaca | -2.821 | 0.122 | 338.991 | -23.138 | <2.00E-16 |
| plantspS.canadensis | -0.916 | 0.151 | 432.847 | -6.079 | 0.000 |
| height_i | 0.062 | 0.015 | 442.355 | 4.167 | 0.000 |
| AM_treatnative:plantspA.syriaca | 2.224 | 0.154 | 434.282 | 14.465 | <2.00E-16 |
| AM_treatnon-native:plantspA.syriaca | 1.971 | 0.154 | 432.458 | 12.781 | <2.00E-16 |
| AM_treatnative:plantspS.canadensis | 1.287 | 0.209 | 432.246 | 6.161 | 0.000 |
| AM_treatnon-native:plantspS.canadensis | 1.049 | 0.210 | 432.352 | 5.004 | 0.000 |
| **Belowground** | | | | | |
| (Intercept) | 0.145 | 0.207 | 121.257 | 0.702 | 0.484169 |
| AM_treatnative | -1.254 | 0.232 | 435.242 | -5.409 | 0.000 |
| AM_treatnon-native | -1.234 | 0.232 | 433.509 | -5.315 | 0.000 |
| plantspA.syriaca | -2.451 | 0.192 | 378.265 | -12.760 | <2.00E-16 |
| plantspS.canadensis | -0.854 | 0.235 | 433.823 | -3.627 | 0.000 |
| height_i | 0.110 | 0.023 | 441.606 | 4.671 | 0.000 |
| AM_treatnative:plantspA.syriaca | 2.464 | 0.240 | 434.866 | 10.259 | <2.00E-16 |
| AM_treatnon-native:plantspA.syriaca | 2.760 | 0.241 | 433.533 | 11.455 | <2.00E-16 |
| AM_treatnative:plantspS.canadensis | 1.268 | 0.326 | 433.375 | 3.886 | 0.000 |
| AM_treatnon-native:plantspS.canadensis | 1.269 | 0.327 | 433.454 | 3.877 | 0.000 |
| **Contrasts** | | | | | |
| **Aboveground** | | | | | |
| A.cannabinum sterile v AMF | -1.125 | 0.129 | 436.77 | -8.748 | <.0001 |
| A.syriaca sterile v AMF | 0.973 | 0.035 | 436.17 | 27.663 | <.0001 |
| S.canadensis sterile v AMF | 0.043 | 0.128 | 436.66 | 0.337 | 0.736 |
| A.cannabinum sterile v native | -1.310 | 0.148 | 437.74 | -8.825 | <.0001 |
| A.syriaca sterile v native | 0.914 | 0.041 | 436.25 | 22.455 | <.0001 |
| S.canadensis sterile v native | -0.023 | 0.148 | 437.72 | -0.154 | 0.877 |
| A.cannabinum sterile v non-native | -0.940 | 0.149 | 436.22 | -6.324 | <.0001 |
| A.syriaca sterile v non-native | 1.032 | 0.041 | 436.19 | 25.288 | <.0001 |
| S.canadensis sterile v non-native | 0.109 | 0.148 | 436.10 | 0.740 | 0.460 |
| A.cannabinum native v non-native | 0.370 | 0.149 | 437.67 | 2.492 | 0.013 |
| A.syriaca native v non-native | 0.118 | 0.041 | 436.38 | 2.868 | 0.004 |
| S.canadensis native v non-native | 0.132 | 0.148 | 437.73 | 0.891 | 0.374 |
| **Belowground** | | | | | |
| A.cannabinum sterile v AMF | -1.244 | 0.201 | 436.61 | -6.192 | <.0001 |
| A.syriaca sterile v AMF | 1.368 | 0.055 | 436.12 | 24.863 | <.0001 |
| S.canadensis sterile v AMF | 0.025 | 0.200 | 436.52 | 0.125 | 0.900 |
| A.cannabinum sterile v native | -1.254 | 0.232 | 437.41 | -5.407 | <.0001 |
| A.syriaca sterile v native | 1.210 | 0.064 | 436.18 | 19.036 | <.0001 |
| S.canadensis sterile v native | 0.014 | 0.232 | 437.40 | 0.062 | 0.950 |
| A.cannabinum sterile v non-native | -1.234 | 0.232 | 436.16 | -5.315 | <.0001 |
| A.syriaca sterile v non-native | 1.526 | 0.064 | 436.14 | 23.909 | <.0001 |
| S.canadensis sterile v non-native | 0.036 | 0.231 | 436.06 | 0.155 | 0.877 |
| A.cannabinum native v non-native | 0.020 | 0.232 | 437.35 | 0.087 | 0.931 |
| A.syriaca native v non-native | 0.316 | 0.064 | 436.27 | 4.929 | <.0001 |
| S.canadensis native v non-native | 0.021 | 0.232 | 437.41 | 0.092 | 0.927 |

**Table S5 | Disease resistance**

Model outputs for disease incidence with associated custom contrasts; shown per plant species.

| **Disease Incidence** | | | | | |
| --- | --- | --- | --- | --- | --- |
| **All species** | | | | | |
| variable | estimate | std. error | df | t value | p value |
| (Intercept) | 3.405 | 0.574 | 109.627 | 5.929 | 0.000 |
| AM_treatnative | -0.959 | 0.612 | 158.457 | -1.568 | 0.119 |
| AM_treatnon-native | -0.563 | 0.616 | 158.013 | -0.913 | 0.363 |
| path_treatpath | 0.720 | 0.616 | 158.316 | 1.169 | 0.244 |
| plantspA.syriaca | -2.280 | 0.634 | 158.057 | -3.595 | 0.000 |
| plantspS.canadensis | -2.216 | 0.633 | 158.053 | -3.504 | 0.001 |
| height_i | -0.059 | 0.086 | 158.757 | -0.691 | 0.491 |
| AM_treatnative:path_treatpath | -0.753 | 0.866 | 158.754 | -0.870 | 0.385 |
| AM_treatnon-native:path_treatpath | -0.775 | 0.869 | 158.009 | -0.892 | 0.374 |
| AM_treatnative:plantspA.syriaca | 1.727 | 0.863 | 157.998 | 2.001 | 0.047 |
| AM_treatnon-native:plantspA.syriaca | 1.582 | 0.870 | 158.011 | 1.818 | 0.071 |
| AM_treatnative:plantspS.canadensis | 0.708 | 0.862 | 157.996 | 0.821 | 0.413 |
| AM_treatnon-native:plantspS.canadensis | 0.168 | 0.868 | 158.006 | 0.194 | 0.847 |
| path_treatpath:plantspA.syriaca | 1.969 | 0.863 | 157.998 | 2.280 | 0.024 |
| path_treatpath:plantspS.canadensis | 0.565 | 0.864 | 157.999 | 0.654 | 0.514 |
| AM_treatnative:path_treatpath:plantspA.syriaca | -0.988 | 1.219 | 157.996 | -0.810 | 0.419 |
| AM_treatnon-native:path_treatpath:plantspA.syriaca | -1.768 | 1.220 | 157.997 | -1.448 | 0.149 |
| AM_treatnative:path_treatpath:plantspS.canadensis | 0.912 | 1.219 | 157.996 | 0.748 | 0.456 |
| AM_treatnon-native:path_treatpath:plantspS.canadensis | 1.446 | 1.221 | 157.998 | 1.184 | 0.238 |
| **Contrasts** | | | | | |
| contrast | estimate | SE | df | t.ratio | p.value |
| A.cannabinum path sterile v not * AM sterile v native | 0.753 | 0.867 | 158.76 | 0.869 | 0.386 |
| A.cannabinum path sterile v not * AM sterile v non-native | 0.775 | 0.869 | 158.01 | 0.892 | 0.374 |
| A.syriaca path sterile v not * AM sterile v native | 1.742 | 0.867 | 158.78 | 2.009 | 0.046 |
| A.syriaca path sterile v not * AM sterile v non-native | 2.543 | 0.864 | 158 | 2.941 | 0.004 |
| S.canadensis path sterile v not * AM sterile v native | -0.158 | 0.867 | 158.78 | -0.183 | 0.855 |
| S.canadensis path sterile v not * AM sterile v non-native | -0.671 | 0.863 | 158 | -0.777 | 0.438 |

**Table S6 | Arbuscular mycorrhizal fungal and pathogen**

Model outputs for above- and belowground biomass with associated custom contrasts.

| **Biomass** | | | | | |
| --- | --- | --- | --- | --- | --- |
| ***Asclepias syriaca*** | | | | | |
| **Aboveground** | | | | | |
| variable | estimate | std. error | df | t value | p value |
| (Intercept) | 3.000 | 0.090 | 39.194 | -25.609 | <2.00E-16 |
| AM_treatnative | 0.913 | 0.042 | 948.802 | 21.630 | <2.00E-16 |
| AM_treatnon-native | 1.032 | 0.042 | 948.317 | 24.390 | <2.00E-16 |
| path_treat19 | 0.013 | 0.053 | 950.011 | 0.253 | 0.800 |
| path_treat35 | 0.020 | 0.054 | 950.018 | 0.382 | 0.703 |
| path_treat87 | 0.053 | 0.051 | 950.046 | 1.054 | 0.292 |
| land_useremnant | 0.052 | 0.024 | 955.945 | 2.177 | 0.030 |
| height_i | 0.059 | 0.011 | 955.981 | 5.609 | 0.000 |
| AM_treatnative:path_treat19 | 0.128 | 0.074 | 948.442 | 1.721 | 0.086 |
| AM_treatnon-native:path_treat19 | 0.018 | 0.076 | 948.295 | 0.233 | 0.816 |
| AM_treatnative:path_treat35 | 0.063 | 0.075 | 948.462 | 0.837 | 0.403 |
| AM_treatnon-native:path_treat35 | 0.151 | 0.075 | 948.296 | 2.008 | 0.045 |
| AM_treatnative:path_treat87 | 0.014 | 0.071 | 949.675 | 0.195 | 0.845 |
| AM_treatnon-native:path_treat87 | -0.004 | 0.072 | 948.298 | -0.058 | 0.954 |
| **Belowground** | | | | | |
| Intercept) | -2.460 | 0.138 | 42.429 | -17.768 | <2.00E-16 |
| AM_treatnative | 1.213 | 0.065 | 947.000 | 18.545 | <2.00E-16 |
| AM_treatnon-native | 1.526 | 0.066 | 946.509 | 23.252 | <2.00E-16 |
| path_treat19 | -0.036 | 0.082 | 948.199 | -0.436 | 0.663 |
| path_treat35 | -0.024 | 0.083 | 948.288 | -0.284 | 0.776 |
| path_treat87 | 0.055 | 0.079 | 948.266 | 0.697 | 0.486 |
| land_useremnant | 0.091 | 0.037 | 953.856 | 2.464 | 0.014 |
| height_i | 0.123 | 0.016 | 953.923 | 7.550 | 0.000 |
| AM_treatnative:path_treat19 | 0.253 | 0.115 | 946.635 | 2.191 | 0.029 |
| AM_treatnon-native:path_treat19 | 0.198 | 0.118 | 946.489 | 1.684 | 0.093 |
| AM_treatnative:path_treat35 | 0.005 | 0.117 | 946.652 | 0.041 | 0.967 |
| AM_treatnon-native:path_treat35 | 0.324 | 0.117 | 946.488 | 2.778 | 0.006 |
| AM_treatnative:path_treat87 | 0.067 | 0.111 | 947.874 | 0.602 | 0.548 |
| AM_treatnon-native:path_treat87 | 0.024 | 0.112 | 946.489 | 0.212 | 0.832 |
| **Contrasts** | | | | | |
| **Aboveground** | | | | | |
| contrast | estimate | SE | df | t ratio | p value |
| AM sterile v native | 3.859 | 0.113 | 949.19 | 34.173 | <.0001 |
| AM sterile v non-native | 4.293 | 0.114 | 949.2 | 37.625 | <.0001 |
| AM native v non-native | 0.434 | 0.113 | 949.25 | 3.837 | 0.000 |
| path sterile v 19 * AM sterile v native | -0.128 | 0.074 | 949.28 | -1.721 | 0.086 |
| path sterile v 35 * AM sterile v native | -0.063 | 0.075 | 949.3 | -0.837 | 0.403 |
| path sterile v 87 * AM sterile v native | -0.014 | 0.071 | 950.38 | -0.195 | 0.845 |
| path sterile v 19 * AM sterile v non-native | -0.018 | 0.076 | 949.15 | -0.233 | 0.816 |
| path sterile v 35 * AM sterile v non-native | -0.151 | 0.075 | 949.15 | -2.008 | 0.045 |
| path sterile v 87 * AM sterile v non-native | 0.004 | 0.072 | 949.15 | 0.058 | 0.954 |
| **Belowground** | | | | | |
| AM sterile v native | 5.175 | 0.175 | 947.2 | 29.595 | <.0001 |
| AM sterile v non-native | 6.652 | 0.177 | 947.23 | 37.530 | <.0001 |
| AM native v non-native | 1.477 | 0.176 | 947.27 | 8.404 | <.0001 |
| path sterile v 19 * AM sterile v native | -0.253 | 0.115 | 947.3 | -2.191 | 0.029 |
| path sterile v 35 * AM sterile v native | -0.005 | 0.117 | 947.31 | -0.041 | 0.967 |
| path sterile v 87 * AM sterile v native | -0.067 | 0.111 | 948.43 | -0.601 | 0.548 |
| path sterile v 19 * AM sterile v non-native | -0.198 | 0.118 | 947.17 | -1.684 | 0.093 |
| path sterile v 35 * AM sterile v non-native | -0.324 | 0.117 | 947.16 | -2.778 | 0.006 |
| path sterile v 87 * AM sterile v non-native | -0.024 | 0.112 | 947.17 | -0.212 | 0.832 |
