# Supplementary material for: Origin matters: mycorrhizal growth response and induced resistance to pathogens depend on mycorrhizal and pathogen source
Source: New Phytol. 2025 Jul 6;248(3):1516–26. doi: 10.1111/nph.70358 (PMC12489296; doi:10.1111/nph.70358)
Supplement: Supplementary file 1 — Fig. S1 Map of sampling sites. Fig. S2 Disease incidence assessment. Fig. S3 Arbuscular mycorrhizal fungal inocula source influences growth response in Asclepias syriaca. Table S1 Pathogenicity growth results. Table S2 Pathogenicity survival results. [file NPH-248-1516-s002.zip › MIRKS_SI_July22025.pdf]

New Phytologist Supporting Information

Article title: Origin matters: mycorrhizal growth response and induced resistance to pathogens depends on mycorrhizal and pathogen source

Authors: *Camille S. Delavaux, Haley Burrill, Robert Menning, Eric B. Duell, Reb L. Bryant, Terra Lubin, James D. Beve*

Article acceptance date: 17 June 2025

Figure S1 | Map of sampling sites

Map of sampling sites, including five remnant and five post-agricultural sites. All sites are located in Kansas, USA (KS) , with the exception of one site in western Missouri, USA (MO).

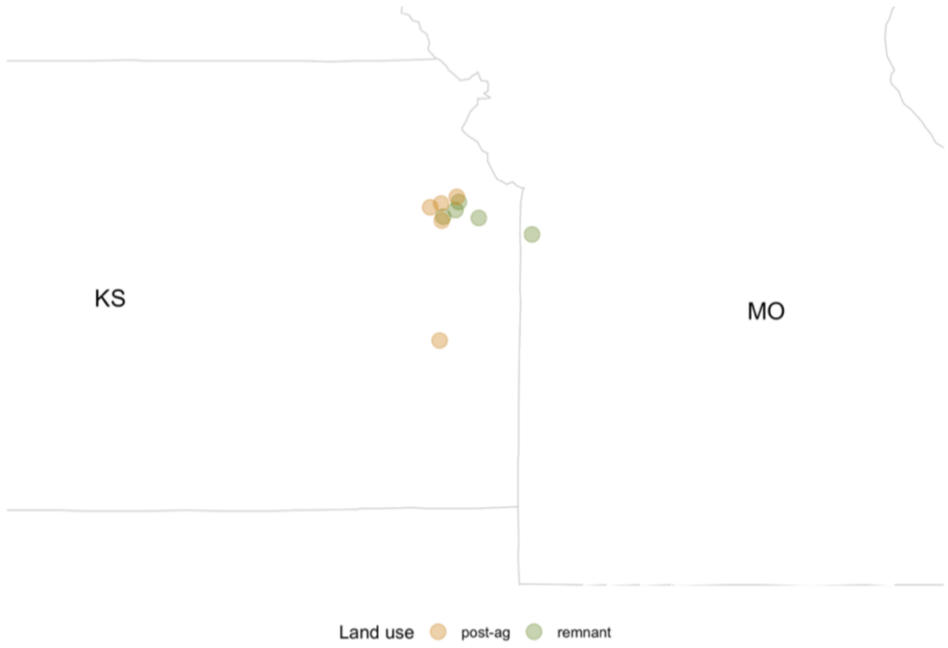

Figure S2 | Disease incidence assessment

Photos of example plant of disease incidence score 4, where there are >2 spots and on multiple leaves.

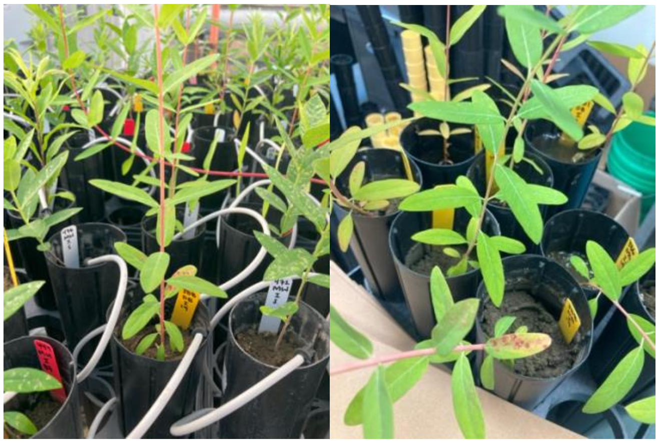

Figure S3 | Arbuscular mycorrhizal fungal inocula source influences growth response in *Asclepias syriaca*

Asclepias syriaca responds positively to addition of arbuscular mycorrhizal fungi (AMF), with biomass significantly higher for both AMF treatments, compared to sterile ($p < 0.0001$). Moreover, *Asclepias syriaca* responds significantly more positively to addition of non-native AMF relative to native AMF ($p < 0.001$). *Apocynum cannabinum* responds negatively to the addition of either native or non-native AMF ($p < 0.001$) and responds marginally significantly more positively to addition of non-native AMF relative to native AMF ($p = 0.013$), while *Solidago canadensis* does not respond to AMF addition ($p = 0.7$). Error bars represent standard errors.

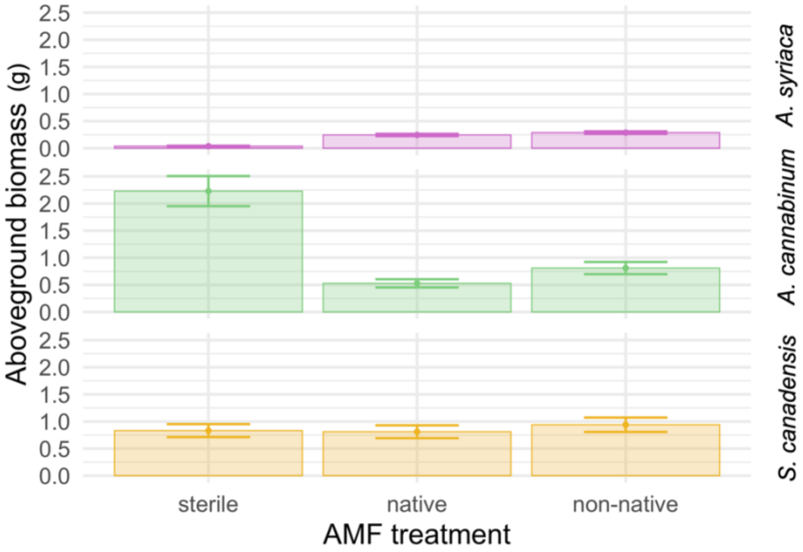

Table S1 | Pathogenicity growth results

Model outputs for pathogenicity tests on above- and belowground biomass; shown per plant species.

**Aboveground**					
Asclepias syriaca					
variable	estimate	std. error	df	t value	p value
(Intercept)	-2.173	0.027	104	-80.511	<2.00E-16
path_treat18	-0.016	0.032	104	-0.486	0.628
path_treat19	-0.027	0.036	104	-0.758	0.450

path_treat23	-0.037	0.029	104	-1.281	0.203
path_treat29	-0.016	0.061	104	-0.257	0.798
path_treat35	-0.024	0.045	104	-0.531	0.596
path_treat45	0.003	0.032	104	0.089	0.929
path_treat57	0.021	0.025	104	0.830	0.409
path_treat58	0.043	0.027	104	1.610	0.110
path_treat59	0.000	0.027	104	0.009	0.993
path_treat61	-0.008	0.030	104	-0.257	0.798
path_treat62	-0.007	0.029	104	-0.235	0.815
path_treat63	-0.007	0.031	104	-0.215	0.831
path_treat64	0.029	0.022	104	1.319	0.190
path_treat73	0.022	0.037	104	0.598	0.551
path_treat74	0.049	0.036	104	1.353	0.179
path_treat76	-0.016	0.032	104	-0.507	0.613
path_treat83	-0.005	0.029	104	-0.173	0.863
path_treat85	0.021	0.025	104	0.852	0.396
path_treat87	-0.051	0.029	104	-1.755	0.082
path_treat92	0.001	0.027	104	0.053	0.958
height_i	0.010	0.007	104	1.402	0.164
Apocynum cannabinum					
(Intercept)	-2.322	0.045	180	-51.251	<2.00E-16
path_treat17	0.080	0.054	182	1.496	0.136
path_treat18	0.065	0.056	174	1.154	0.250
path_treat19	-0.001	0.061	178	-0.014	0.989
path_treat23	0.068	0.056	162	1.207	0.229
path_treat29	-0.027	0.048	178	-0.569	0.570
path_treat30	-0.057	0.068	168	-0.835	0.405
path_treat33	-0.064	0.063	175	-1.012	0.313
path_treat35	0.072	0.048	179	1.517	0.131
path_treat38	-0.086	0.062	181	-1.377	0.170
path_treat40	-0.045	0.063	173	-0.715	0.476
path_treat41	-0.017	0.069	178	-0.239	0.812
path_treat42	-0.026	0.061	175	-0.421	0.674
path_treat43	-0.069	0.069	178	-0.997	0.320
path_treat44	-0.076	0.063	177	-1.212	0.227
path_treat45	-0.050	0.044	173	-1.147	0.253
path_treat46	-0.019	0.062	182	-0.307	0.760
path_treat57	0.237	0.054	182	4.377	0.000
path_treat58	0.119	0.054	182	2.216	0.028

path_treat59	0.187	0.055	171	3.401	0.001
path_treat64	0.018	0.061	178	0.296	0.768
path_treat73	0.133	0.061	174	2.181	0.031
path_treat74	0.166	0.069	181	2.414	0.017
path_treat76	0.002	0.061	178	0.040	0.968
path_treat83	0.077	0.057	182	1.336	0.183
path_treat85	0.033	0.053	182	0.623	0.534
path_treat87	0.074	0.057	181	1.305	0.194
path_treat92	0.071	0.042	176	1.691	0.093
height_i	0.053	0.014	176	3.870	0.000
Solidago canadensis					
(Intercept)	-2.278	0.017	86	-130.535	<2.00E-16
path_treat17	0.041	0.028	86	1.439	0.154
path_treat18	0.027	0.031	86	0.877	0.383
path_treat19	0.112	0.027	86	4.099	0.000
path_treat23	0.009	0.027	86	0.333	0.740
path_treat29	-0.005	0.034	86	-0.144	0.886
path_treat45	0.002	0.026	86	0.094	0.925
path_treat57	0.056	0.025	86	2.290	0.024
path_treat58	0.013	0.026	86	0.478	0.634
path_treat59	0.049	0.026	86	1.859	0.067
path_treat64	-0.029	0.036	86	-0.808	0.421
path_treat68	-0.017	0.056	86	-0.297	0.767
path_treat70	-0.002	0.034	86	-0.053	0.958
path_treat72	-0.021	0.030	86	-0.694	0.490
path_treat74	-0.016	0.031	86	-0.536	0.593
path_treat76	0.005	0.028	86	0.161	0.872
path_treat83	0.033	0.025	86	1.322	0.190
path_treat85	0.090	0.035	86	2.575	0.012
path_treat87	0.055	0.029	86	1.855	0.067
path_treat92	0.020	0.026	86	0.768	0.445
height_i	0.020	0.016	86	1.278	0.205
Eupatorium altissimum					
(Intercept)	-2.265	0.025	141	-92.089	<2.00E-16
path_treat1	-0.014	0.041	141	-0.349	0.728
path_treat10	-0.003	0.037	141	-0.089	0.929
path_treat12	-0.018	0.041	141	-0.436	0.664
path_treat14	-0.075	0.041	141	-1.857	0.065
path_treat16	0.029	0.038	141	0.776	0.439

path_treat17	0.070	0.028	141	2.519	0.013
path_treat18	0.021	0.029	141	0.713	0.477
path_treat19	0.010	0.030	141	0.341	0.734
path_treat2	-0.053	0.040	141	-1.319	0.189
path_treat20	0.028	0.041	141	0.677	0.500
path_treat21	0.002	0.047	141	0.033	0.974
path_treat22	-0.003	0.041	141	-0.072	0.943
path_treat23	-0.016	0.030	141	-0.541	0.590
path_treat25	-0.014	0.077	141	-0.182	0.856
path_treat26	0.005	0.041	141	0.123	0.902
path_treat29	-0.072	0.077	141	-0.934	0.352
path_treat35	-0.029	0.046	141	-0.629	0.530
path_treat45	-0.057	0.078	141	-0.727	0.469
path_treat57	0.020	0.034	141	0.577	0.565
path_treat58	0.036	0.032	141	1.135	0.258
path_treat59	0.009	0.037	141	0.254	0.800
path_treat64	0.000	0.034	141	-0.004	0.996
path_treat7	-0.035	0.037	141	-0.943	0.347
path_treat73	0.001	0.056	141	0.020	0.984
path_treat74	-0.029	0.077	141	-0.372	0.710
path_treat76	0.051	0.041	141	1.264	0.208
path_treat8	-0.041	0.037	141	-1.117	0.266
path_treat83	0.010	0.056	141	0.177	0.860
path_treat85	0.018	0.046	141	0.381	0.704
path_treat87	-0.039	0.037	141	-1.066	0.288
path_treat9	-0.012	0.037	141	-0.333	0.739
path_treat92	0.007	0.034	141	0.212	0.832
height_i	0.053	0.013	141	3.995	0.000
Vernonia fasciculata					
(Intercept)	-2.285	0.029	144	-79.829	<2.00E-16
path_treat17	-0.012	0.037	149	-0.334	0.739
path_treat18	0.002	0.039	148	0.041	0.967
path_treat19	0.120	0.039	148	3.050	0.003
path_treat23	-0.005	0.037	149	-0.137	0.891
path_treat29	0.003	0.065	149	0.043	0.966
path_treat35	-0.033	0.091	149	-0.367	0.714
path_treat45	0.051	0.047	149	1.081	0.282
path_treat47	0.000	0.044	148	0.000	1.000
path_treat48	-0.033	0.044	146	-0.754	0.452

path_treat49	-0.006	0.054	149	-0.103	0.918
path_treat51	-0.017	0.048	137	-0.359	0.720
path_treat53	-0.028	0.043	149	-0.654	0.514
path_treat55	0.010	0.045	132	0.218	0.828
path_treat56	0.001	0.048	124	0.025	0.980
path_treat57	0.031	0.032	147	0.985	0.326
path_treat58	0.004	0.032	149	0.130	0.896
path_treat59	0.044	0.030	148	1.460	0.146
path_treat64	0.017	0.037	149	0.475	0.636
path_treat73	0.026	0.047	140	0.545	0.587
path_treat74	-0.002	0.054	149	-0.043	0.966
path_treat76	-0.010	0.047	149	-0.211	0.833
path_treat83	0.013	0.037	148	0.359	0.720
path_treat85	-0.007	0.043	149	-0.157	0.875
path_treat87	-0.008	0.037	149	-0.212	0.833
path_treat92	-0.010	0.040	149	-0.259	0.796
height_i	0.045	0.013	146	3.486	0.001
**Belowground**					
Asclepias syriaca					
(Intercept)	-2.060	0.044	104	-47.092	<2.00E-16
path_treat17	0.097	0.052	101	1.869	0.065
path_treat18	-0.039	0.051	95	-0.771	0.443
path_treat19	0.047	0.058	103	0.804	0.424
path_treat23	-0.019	0.047	105	-0.407	0.685
path_treat29	-0.087	0.097	101	-0.891	0.375
path_treat35	-0.092	0.072	105	-1.281	0.203
path_treat45	0.039	0.050	92	0.772	0.442
path_treat57	-0.001	0.040	99	-0.023	0.981
path_treat58	0.079	0.043	102	1.849	0.067
path_treat59	0.020	0.043	101	0.460	0.647
path_treat61	0.030	0.049	104	0.622	0.536
path_treat62	-0.015	0.046	99	-0.322	0.748
path_treat63	0.047	0.055	105	0.864	0.389
path_treat64	0.005	0.035	105	0.152	0.879
path_treat73	-0.066	0.058	98	-1.134	0.260
path_treat74	0.039	0.058	100	0.678	0.500
path_treat76	-0.047	0.051	100	-0.926	0.357
path_treat83	-0.029	0.050	81	-0.593	0.555
path_treat85	0.034	0.041	105	0.844	0.401

path_treat87	-0.082	0.047	103	-1.722	0.088
path_treat92	0.000	0.043	105	-0.006	0.995
height_i	0.014	0.011	104	1.301	0.196
Apocynum cannabinum					
(Intercept)	-2.238	0.030	174	-74.252	<2.00E-16
path_treat17	0.038	0.036	173	1.066	0.288
path_treat18	0.033	0.037	160	0.890	0.375
path_treat19	-0.046	0.041	169	-1.138	0.257
path_treat23	0.038	0.037	145	1.031	0.304
path_treat29	-0.021	0.032	173	-0.675	0.501
path_treat30	-0.027	0.045	153	-0.601	0.549
path_treat33	-0.064	0.043	179	-1.486	0.139
path_treat35	-0.011	0.032	175	-0.348	0.728
path_treat38	-0.092	0.042	180	-2.208	0.029
path_treat40	-0.059	0.043	181	-1.389	0.167
path_treat41	-0.011	0.046	172	-0.230	0.819
path_treat42	-0.026	0.040	166	-0.638	0.524
path_treat43	-0.015	0.047	181	-0.323	0.747
path_treat44	-0.044	0.042	181	-1.033	0.303
path_treat45	-0.025	0.029	159	-0.857	0.393
path_treat46	-0.051	0.042	180	-1.212	0.227
path_treat57	0.131	0.036	178	3.608	0.000
path_treat58	0.030	0.036	178	0.831	0.407
path_treat59	0.140	0.037	172	3.739	0.000
path_treat64	0.027	0.041	170	0.669	0.504
path_treat73	0.110	0.040	163	2.733	0.007
path_treat74	0.198	0.046	180	4.277	0.000
path_treat76	-0.014	0.041	170	-0.336	0.737
path_treat83	0.074	0.038	180	1.919	0.057
path_treat85	0.007	0.036	181	0.200	0.841
path_treat87	-0.002	0.038	181	-0.052	0.959
path_treat92	0.026	0.028	165	0.947	0.345
height_i	0.042	0.009	166	4.578	0.000
Solidago canadensis					
(Intercept)	-2.284	0.014	61	-160.142	<2.00E-16
path_treat17	0.018	0.021	67	0.861	0.393
path_treat18	0.014	0.021	67	0.639	0.525
path_treat19	0.018	0.019	67	0.937	0.352
path_treat23	0.016	0.020	67	0.814	0.419

path_treat29	-0.002	0.028	66	-0.074	0.941
path_treat35	0.015	0.038	64	0.391	0.697
path_treat45	0.004	0.018	62	0.248	0.805
path_treat57	0.003	0.017	67	0.158	0.875
path_treat58	0.022	0.019	67	1.131	0.262
path_treat59	0.000	0.018	67	0.015	0.988
path_treat64	-0.010	0.038	66	-0.271	0.787
path_treat68	-0.008	0.038	66	-0.220	0.827
path_treat70	-0.018	0.038	66	-0.476	0.635
path_treat74	-0.015	0.028	66	-0.525	0.601
path_treat76	0.005	0.020	66	0.255	0.800
path_treat83	0.016	0.021	66	0.767	0.446
path_treat85	0.073	0.022	67	3.353	0.001
path_treat87	0.050	0.020	65	2.453	0.017
path_treat92	-0.001	0.020	66	-0.044	0.965
height_i	0.010	0.012	64	0.798	0.428
Eupatorium altissimum					
(Intercept)	-2.273	0.011	134	-201.621	<2.00E-16
path_treat1	-0.014	0.018	134	-0.755	0.451
path_treat10	-0.005	0.017	134	-0.292	0.771
path_treat12	0.016	0.018	134	0.892	0.374
path_treat14	-0.014	0.018	134	-0.796	0.428
path_treat16	0.000	0.017	134	-0.022	0.983
path_treat17	0.000	0.012	134	-0.033	0.974
path_treat18	0.033	0.014	134	2.437	0.016
path_treat19	0.003	0.013	134	0.255	0.799
path_treat2	-0.013	0.020	134	-0.648	0.518
path_treat20	-0.001	0.021	134	-0.035	0.972
path_treat21	-0.015	0.021	134	-0.715	0.476
path_treat22	0.019	0.018	134	1.042	0.299
path_treat23	-0.026	0.014	134	-1.851	0.066
path_treat25	-0.009	0.034	134	-0.277	0.782
path_treat26	-0.007	0.018	134	-0.412	0.681
path_treat29	-0.009	0.034	134	-0.277	0.782
path_treat35	-0.014	0.021	134	-0.701	0.484
path_treat45	-0.028	0.034	134	-0.820	0.414
path_treat57	-0.001	0.015	134	-0.091	0.928
path_treat58	0.029	0.015	134	1.948	0.054
path_treat59	0.008	0.016	134	0.506	0.614

path_treat64	-0.009	0.015	134	-0.605	0.546
path_treat7	-0.012	0.016	134	-0.744	0.458
path_treat73	-0.003	0.025	134	-0.112	0.911
path_treat74	-0.027	0.034	134	-0.795	0.428
path_treat76	0.004	0.018	134	0.231	0.817
path_treat8	0.023	0.016	134	1.425	0.156
path_treat83	-0.031	0.025	134	-1.276	0.204
path_treat85	0.022	0.020	134	1.094	0.276
path_treat87	-0.023	0.018	134	-1.273	0.205
path_treat9	-0.003	0.016	134	-0.186	0.853
path_treat92	-0.001	0.015	134	-0.033	0.974
height_i	0.015	0.006	134	2.560	0.012
Vernonia fasciculata					
(Intercept)	-2.271	0.033	139	-69.465	<2.00E-16
path_treat17	0.054	0.042	148	1.274	0.205
path_treat18	-0.021	0.045	145	-0.464	0.643
path_treat19	-0.024	0.045	144	-0.544	0.587
path_treat23	-0.010	0.042	147	-0.244	0.808
path_treat29	0.032	0.074	148	0.435	0.665
path_treat35	-0.023	0.103	146	-0.226	0.821
path_treat45	0.402	0.054	148	7.488	0.000
path_treat47	0.006	0.050	146	0.126	0.900
path_treat48	0.031	0.050	141	0.618	0.538
path_treat49	0.013	0.061	148	0.214	0.831
path_treat51	-0.004	0.054	126	-0.080	0.936
path_treat53	0.017	0.048	148	0.355	0.723
path_treat55	-0.013	0.051	118	-0.264	0.793
path_treat56	-0.011	0.056	108	-0.190	0.850
path_treat57	-0.015	0.036	142	-0.419	0.676
path_treat58	-0.005	0.036	148	-0.126	0.900
path_treat59	0.009	0.036	148	0.260	0.795
path_treat64	0.009	0.042	147	0.203	0.839
path_treat73	0.013	0.054	130	0.235	0.815
path_treat74	0.000	0.061	148	-0.004	0.997
path_treat76	0.039	0.054	148	0.730	0.467
path_treat83	0.065	0.042	144	1.551	0.123
path_treat85	0.041	0.048	148	0.840	0.402
path_treat87	0.007	0.042	148	0.176	0.860
path_treat92	0.048	0.045	147	1.053	0.294

height_i	0.032	0.015	143	2.163	0.032

Table S2 | Pathogenicity survival results

Model outputs for pathogenicity tests on survival; shown per plant species.

**Survival**				
Asclepias syriaca				
variable	estimate	std. error	z value	p value
(Intercept)	-0.631	0.866	-0.728	0.466
path_treat17	-1.036	0.879	-1.179	0.238
path_treat18	-1.083	0.884	-1.225	0.221
path_treat19	-1.378	0.867	-1.591	0.112
path_treat23	0.001	0.937	0.002	0.999
path_treat29	-2.061	1.200	-1.717	0.086
path_treat35	-1.023	1.013	-1.010	0.312
path_treat45	-0.594	0.856	-0.694	0.488
path_treat57	19.030	8350.000	0.002	0.998
path_treat58	0.746	1.159	0.644	0.520
path_treat59	0.698	1.157	0.603	0.547
path_treat61	18.030	8202.000	0.002	0.998
path_treat62	18.660	9163.000	0.002	0.998
path_treat63	16.320	3741.000	0.004	0.997
path_treat64	0.462	0.875	0.528	0.597
path_treat73	-0.185	1.012	-0.183	0.855
path_treat74	-0.270	1.023	-0.264	0.792
path_treat76	0.693	1.202	0.577	0.564
path_treat83	0.030	0.929	0.032	0.974
path_treat85	19.430	11140.000	0.002	0.999
path_treat87	0.151	0.940	0.161	0.872
path_treat92	0.917	1.157	0.792	0.428
height_i	0.470	0.233	2.020	0.043
Apocynum cannabinum				
(Intercept)	-0.399	1.172	-0.340	0.734
path_treat17	21.460	81950.000	0.000	1.000
path_treat18	-1.272	1.266	-1.005	0.315
path_treat19	-1.606	1.034	-1.553	0.120
path_treat23	-1.409	1.282	-1.099	0.272
path_treat29	-0.916	1.248	-0.734	0.463

path_treat30	-2.040	1.356	-1.505	0.132
path_treat33	21.080	91820.000	0.000	1.000
path_treat35	-0.705	1.229	-0.574	0.566
path_treat38	21.390	88260.000	0.000	1.000
path_treat40	21.980	86010.000	0.000	1.000
path_treat41	-0.093	1.296	-0.072	0.943
path_treat42	22.730	107700.000	0.000	1.000
path_treat43	21.330	99100.000	0.000	1.000
path_treat44	21.240	73750.000	0.000	1.000
path_treat45	-0.140	1.218	-0.115	0.909
path_treat46	21.800	77060.000	0.000	1.000
path_treat57	21.680	105000.000	0.000	1.000
path_treat58	23.880	133000.000	0.000	1.000
path_treat59	21.330	87130.000	0.000	1.000
path_treat64	-2.130	1.072	-1.986	0.047
path_treat73	21.390	93070.000	0.000	1.000
path_treat74	-1.807	1.321	-1.368	0.171
path_treat76	22.100	118600.000	0.000	1.000
path_treat83	-0.844	1.237	-0.683	0.495
path_treat85	22.450	95150.000	0.000	1.000
path_treat87	-0.442	1.239	-0.356	0.722
path_treat92	28.660	1709000.000	0.000	1.000
height_i	1.071	0.438	2.443	0.015
Solidago canadensis				
(Intercept)	-3.711	0.841	-4.413	0.000
path_treat17	1.719	1.641	1.047	0.295
path_treat18	-1.030	1.280	-0.805	0.421
path_treat19	2.007	1.862	1.078	0.281
path_treat23	1.307	1.627	0.803	0.422
path_treat29	0.804	1.296	0.620	0.535
path_treat35	-1.419	1.794	-0.791	0.429
path_treat45	3.055	1.502	2.034	0.042
path_treat57	16.800	1844.000	0.009	0.993
path_treat58	2.470	1.750	1.411	0.158
path_treat59	2.237	1.621	1.380	0.168
path_treat64	-1.389	1.282	-1.083	0.279
path_treat68	-1.193	1.883	-0.633	0.526
path_treat70	1.837	1.448	1.269	0.204
path_treat72	2.426	1.549	1.566	0.117

path_treat73	-22.130	67330.000	0.000	1.000
path_treat74	1.221	1.014	1.204	0.229
path_treat75	-6.164	16.500	-0.374	0.709
path_treat76	1.669	0.970	1.721	0.085
path_treat83	1.242	1.252	0.992	0.321
path_treat84	-22.310	320100.000	0.000	1.000
path_treat85	-2.053	1.515	-1.355	0.175
path_treat86	-9.884	16.490	-0.599	0.549
path_treat87	-0.474	1.263	-0.375	0.708
path_treat90	-91.980	30010000.000	0.000	1.000
path_treat92	2.819	1.850	1.524	0.128
height_i	5.018	1.108	4.527	0.000
Eupatorium altissimum				
(Intercept)	-2.280	0.663	-3.440	0.001
path_treat1	0.108	1.198	0.090	0.928
path_treat10	25.590	163000.000	0.000	1.000
path_treat12	1.516	1.206	1.256	0.209
path_treat14	0.830	1.218	0.681	0.496
path_treat16	26.500	215600.000	0.000	1.000
path_treat17	1.203	0.893	1.348	0.178
path_treat18	1.240	0.871	1.424	0.154
path_treat19	0.738	0.774	0.954	0.340
path_treat2	0.315	1.192	0.265	0.791
path_treat20	1.443	1.241	1.163	0.245
path_treat21	1.201	1.092	1.100	0.271
path_treat22	1.648	1.244	1.324	0.185
path_treat23	0.576	0.862	0.668	0.504
path_treat25	-0.707	1.275	-0.554	0.579
path_treat26	1.331	1.250	1.065	0.287
path_treat29	-1.966	1.214	-1.619	0.105
path_treat35	0.215	1.096	0.196	0.844
path_treat45	-1.933	1.161	-1.665	0.096
path_treat57	31.340	5050000.000	0.000	1.000
path_treat58	27.800	727500.000	0.000	1.000
path_treat59	0.012	0.968	0.012	0.991
path_treat64	1.074	1.315	0.816	0.414
path_treat7	25.200	157900.000	0.000	1.000
path_treat73	-0.262	1.036	-0.253	0.801
path_treat74	-1.839	1.232	-1.493	0.135

path_treat76	1.150	1.440	0.798	0.425
path_treat8	25.490	287400.000	0.000	1.000
path_treat83	-1.473	0.969	-1.520	0.129
path_treat85	-1.049	0.951	-1.103	0.270
path_treat87	0.703	1.015	0.693	0.489
path_treat9	24.700	155100.000	0.000	1.000
path_treat92	1.604	1.179	1.361	0.174
height_i	2.258	0.442	5.114	0.000
Vernonia fasciculata				
(Intercept)	0.692	1.051	0.659	0.510
path_treat17	23.777	762.728	0.031	0.975
path_treat18	-0.616	1.212	-0.508	0.611
path_treat19	-0.612	1.216	-0.504	0.614
path_treat23	23.663	815.240	0.029	0.977
path_treat29	-2.813	1.076	-2.614	0.009
path_treat35	-3.327	1.252	-2.658	0.008
path_treat45	-1.939	0.949	-2.044	0.041
path_treat47	22.808	836.386	0.027	0.978
path_treat48	23.129	836.386	0.028	0.978
path_treat49	-2.154	1.086	-1.983	0.047
path_treat51	-0.972	1.258	-0.772	0.440
path_treat53	23.824	812.670	0.029	0.977
path_treat55	23.931	844.814	0.028	0.977
path_treat56	-0.090	1.331	-0.068	0.946
path_treat57	0.074	1.182	0.063	0.950
path_treat58	-0.610	0.944	-0.646	0.518
path_treat59	25.524	812.670	0.031	0.975
path_treat64	24.196	832.807	0.029	0.977
path_treat73	-0.573	1.258	-0.456	0.648
path_treat74	-1.662	1.069	-1.556	0.120
path_treat76	-0.500	1.260	-0.397	0.691
path_treat83	23.957	911.947	0.026	0.979
path_treat85	-1.439	1.007	-1.429	0.153
path_treat87	23.761	1044.086	0.023	0.982
path_treat92	-0.636	1.218	-0.522	0.602
height_i	0.836	0.513	1.630	0.103

Table S3 | List of all tested fungal and oomycete taxa, field metadata, and associated sequences

Comprehensive list of tested field-collected fungi and oomycete tested in pathogenicity tests and associated metadata, including original population type (R is remnant, OF is old field, or post-agricultural), whether the isolate was sequences, putative species, what experiment it was included in, and FUNGuild derived guild. Uploaded as separate excel file.

Table S4 | Arbuscular mycorrhizal fungal growth and survival

Model outputs for above- and belowground biomass and survival with associated custom contrasts; shown per plant species.

**Aboveground**					
variable	estimate	std. error	df	t value	p value
(Intercept)	0.524	0.130	149.000	4.037	0.000
AM_treatnative	-1.310	0.148	434.788	-8.830	<2.00E-16
AM_treatnon-native	-0.940	0.149	432.426	-6.324	0.000
plantspA.syriaca	-2.821	0.122	338.991	-23.138	<2.00E-16
plantspS.canadensis	-0.916	0.151	432.847	-6.079	0.000
height_i	0.062	0.015	442.355	4.167	0.000
AM_treatnative:plantspA.syriaca	2.224	0.154	434.282	14.465	<2.00E-16
AM_treatnon-native:plantspA.syriaca	1.971	0.154	432.458	12.781	<2.00E-16
AM_treatnative:plantspS.canadensis	1.287	0.209	432.246	6.161	0.000
AM_treatnon-native:plantspS.canadensis	1.049	0.210	432.352	5.004	0.000
**Belowground**					
(Intercept)	0.145	0.207	121.257	0.702	0.484169
AM_treatnative	-1.254	0.232	435.242	-5.409	0.000
AM_treatnon-native	-1.234	0.232	433.509	-5.315	0.000

plantspA.syriaca	-2.451	0.192	378.265	-12.760	<2.00E-16
plantspS.canadensis	-0.854	0.235	433.823	-3.627	0.000
height_i	0.110	0.023	441.606	4.671	0.000
AM_treatnative:plantspA.syriaca	2.464	0.240	434.866	10.259	<2.00E-16
AM_treatnon-native:plantspA.syriaca	2.760	0.241	433.533	11.455	<2.00E-16
AM_treatnative:plantspS.canadensis	1.268	0.326	433.375	3.886	0.000
AM_treatnon-native:plantspS.canadensis	1.269	0.327	433.454	3.877	0.000
**Contrasts**					
**Aboveground**					
A.cannabinum sterile v AMF	-1.125	0.129	436.77	-8.748	<.0001
A.syriaca sterile v AMF	0.973	0.035	436.17	27.663	<.0001
S.canadensis sterile v AMF	0.043	0.128	436.66	0.337	0.736
A.cannabinum sterile v native	-1.310	0.148	437.74	-8.825	<.0001
A.syriaca sterile v native	0.914	0.041	436.25	22.455	<.0001
S.canadensis sterile v native	-0.023	0.148	437.72	-0.154	0.877
A.cannabinum sterile v non-native	-0.940	0.149	436.22	-6.324	<.0001
A.syriaca sterile v non-native	1.032	0.041	436.19	25.288	<.0001
S.canadensis sterile v non-native	0.109	0.148	436.10	0.740	0.460
A.cannabinum native v non-native	0.370	0.149	437.67	2.492	0.013
A.syriaca native v non-native	0.118	0.041	436.38	2.868	0.004
S.canadensis native v non-native	0.132	0.148	437.73	0.891	0.374
**Belowground**					
A.cannabinum sterile v AMF	-1.244	0.201	436.61	-6.192	<.0001
A.syriaca sterile v AMF	1.368	0.055	436.12	24.863	<.0001
S.canadensis sterile v AMF	0.025	0.200	436.52	0.125	0.900
A.cannabinum sterile v native	-1.254	0.232	437.41	-5.407	<.0001
A.syriaca sterile v native	1.210	0.064	436.18	19.036	<.0001
S.canadensis sterile v native	0.014	0.232	437.40	0.062	0.950
A.cannabinum sterile v non-native	-1.234	0.232	436.16	-5.315	<.0001
A.syriaca sterile v non-native	1.526	0.064	436.14	23.909	<.0001
S.canadensis sterile v non-native	0.036	0.231	436.06	0.155	0.877
A.cannabinum native v non-native	0.020	0.232	437.35	0.087	0.931
A.syriaca native v non-native	0.316	0.064	436.27	4.929	<.0001
S.canadensis native v non-native	0.021	0.232	437.41	0.092	0.927

Table S5 | Disease resistance

Model outputs for disease incidence with associated custom contrasts; shown per plant species.

Disease Incidence					
**All species**					
variable	estimate	std. error	df	t value	p value
(Intercept)	3.405	0.574	109.627	5.929	0.000
AM_treatnative	-0.959	0.612	158.457	-1.568	0.119
AM_treatnon-native	-0.563	0.616	158.013	-0.913	0.363
path_treatpath	0.720	0.616	158.316	1.169	0.244
plantspA.syriaca	-2.280	0.634	158.057	-3.595	0.000
plantspS.canadensis	-2.216	0.633	158.053	-3.504	0.001
height_i	-0.059	0.086	158.757	-0.691	0.491
AM_treatnative:path_treatpath	-0.753	0.866	158.754	-0.870	0.385
AM_treatnon-native:path_treatpath	-0.775	0.869	158.009	-0.892	0.374
AM_treatnative:plantspA.syriaca	1.727	0.863	157.998	2.001	0.047
AM_treatnon-native:plantspA.syriaca	1.582	0.870	158.011	1.818	0.071
AM_treatnative:plantspS.canadensis	0.708	0.862	157.996	0.821	0.413
AM_treatnon-native:plantspS.canadensis	0.168	0.868	158.006	0.194	0.847
path_treatpath:plantspA.syriaca	1.969	0.863	157.998	2.280	0.024
path_treatpath:plantspS.canadensis	0.565	0.864	157.999	0.654	0.514
AM_treatnative:path_treatpath:plantspA.syriaca	-0.988	1.219	157.996	-0.810	0.419
AM_treatnon-native:path_treatpath:plantspA.syriaca	-1.768	1.220	157.997	-1.448	0.149

AM_treatnative:path_treatpath:plantspS.canadensis	0.912	1.219	157.996	0.748	0.456
AM_treatnon-native:path_treatpath:plantspS.canadensis	1.446	1.221	157.998	1.184	0.238
**Contrasts**					
contrast	estimate	SE	df	t.ratio	p.value
A.cannabinum path sterile v not * AM sterile v native	0.753	0.867	158.76	0.869	0.386
A.cannabinum path sterile v not * AM sterile v non-native	0.775	0.869	158.01	0.892	0.374
A.syriaca path sterile v not * AM sterile v native	1.742	0.867	158.78	2.009	0.046
A.syriaca path sterile v not * AM sterile v non-native	2.543	0.864	158	2.941	0.004
S.canadensis path sterile v not * AM sterile v native	-0.158	0.867	158.78	-0.183	0.855
S.canadensis path sterile v not * AM sterile v non-native	-0.671	0.863	158	-0.777	0.438

Table S6 | Arbuscular mycorrhizal fungal and pathogen

Model outputs for above- and belowground biomass with associated custom contrasts.

**Biomass**					
Asclepias syriaca					
**Aboveground**					
variable	estimate	std. error	df	t value	p value
(Intercept)	3.000	0.090	39.194	-25.609	<2.00E-16
AM_treatnative	0.913	0.042	948.802	21.630	<2.00E-16
AM_treatnon-native	1.032	0.042	948.317	24.390	<2.00E-16
path_treat19	0.013	0.053	950.011	0.253	0.800
path_treat35	0.020	0.054	950.018	0.382	0.703
path_treat87	0.053	0.051	950.046	1.054	0.292
land_useremnant	0.052	0.024	955.945	2.177	0.030
height_i	0.059	0.011	955.981	5.609	0.000

AM_treatnative:path_treat19	0.128	0.074	948.442	1.721	0.086
AM_treatnon-native:path_treat19	0.018	0.076	948.295	0.233	0.816
AM_treatnative:path_treat35	0.063	0.075	948.462	0.837	0.403
AM_treatnon-native:path_treat35	0.151	0.075	948.296	2.008	0.045
AM_treatnative:path_treat87	0.014	0.071	949.675	0.195	0.845
AM_treatnon-native:path_treat87	-0.004	0.072	948.298	-0.058	0.954
**Belowground**					
Intercept)	-2.460	0.138	42.429	-17.768	<2.00E-16
AM_treatnative	1.213	0.065	947.000	18.545	<2.00E-16
AM_treatnon-native	1.526	0.066	946.509	23.252	<2.00E-16
path_treat19	-0.036	0.082	948.199	-0.436	0.663
path_treat35	-0.024	0.083	948.288	-0.284	0.776
path_treat87	0.055	0.079	948.266	0.697	0.486
land_useremnant	0.091	0.037	953.856	2.464	0.014
height_i	0.123	0.016	953.923	7.550	0.000
AM_treatnative:path_treat19	0.253	0.115	946.635	2.191	0.029
AM_treatnon-native:path_treat19	0.198	0.118	946.489	1.684	0.093
AM_treatnative:path_treat35	0.005	0.117	946.652	0.041	0.967
AM_treatnon-native:path_treat35	0.324	0.117	946.488	2.778	0.006
AM_treatnative:path_treat87	0.067	0.111	947.874	0.602	0.548
AM_treatnon-native:path_treat87	0.024	0.112	946.489	0.212	0.832
**Contrasts**					
**Aboveground**					
contrast	estimate	SE	df	t ratio	p value

AM sterile v native	3.859	0.113	949.19	34.173	<.0001
AM sterile v non-native	4.293	0.114	949.2	37.625	<.0001
AM native v non-native	0.434	0.113	949.25	3.837	0.000
path sterile v 19 * AM sterile v native	-0.128	0.074	949.28	-1.721	0.086
path sterile v 35 * AM sterile v native	-0.063	0.075	949.3	-0.837	0.403
path sterile v 87 * AM sterile v native	-0.014	0.071	950.38	-0.195	0.845
path sterile v 19 * AM sterile v non-native	-0.018	0.076	949.15	-0.233	0.816
path sterile v 35 * AM sterile v non-native	-0.151	0.075	949.15	-2.008	0.045
path sterile v 87 * AM sterile v non-native	0.004	0.072	949.15	0.058	0.954
**Belowground**					
AM sterile v native	5.175	0.175	947.2	29.595	<.0001
AM sterile v non-native	6.652	0.177	947.23	37.530	<.0001
AM native v non-native	1.477	0.176	947.27	8.404	<.0001
path sterile v 19 * AM sterile v native	-0.253	0.115	947.3	-2.191	0.029
path sterile v 35 * AM sterile v native	-0.005	0.117	947.31	-0.041	0.967
path sterile v 87 * AM sterile v native	-0.067	0.111	948.43	-0.601	0.548
path sterile v 19 * AM sterile v non-native	-0.198	0.118	947.17	-1.684	0.093
path sterile v 35 * AM sterile v non-native	-0.324	0.117	947.16	-2.778	0.006
path sterile v 87 * AM sterile v non-native	-0.024	0.112	947.17	-0.212	0.832
